# The role of the Helmholtz potential on electrocatalytic activity

**DOI:** 10.1038/s41467-026-70980-5

**Published:** 2026-03-28

**Authors:** Arsène Chemin, Louis Godeffroy, David Amans, Tristan Petit

**Affiliations:** 1 Université Claude Bernard Lyon 1, CNRS, Institut Lumière Matière, UMR5306, F-69100, Villeurbanne, France; 2 Helmholtz-Zentrum Berlin für Materialien und Energie GmbH, Nanoscale Solid-Liquid Interfaces, Berlin, Germany

**Keywords:** Electrocatalysis, Energy, Chemical physics, Surfaces, interfaces and thin films

## Abstract

The electrification of the chemical industry is required for a rapid reduction of its carbon footprint and necessitates sustainable and highly active electrocatalysts. New concepts, such as the entropy of the electrolyte at the interface, are emerging as critical descriptors of electrocatalytic activity. However, a theoretical understanding of these properties of the electrochemical interface is still missing. Here, we include the electronic equilibrium at the electrode-electrolyte interface in the Butler-Volmer formalism for metal and metal/semiconductor electrodes. We demonstrate, using experimental data on the hydrogen evolution reaction from the literature, that the electrochemical reaction kinetics are not only governed by the Sabatier principle, but also by the Helmholtz potential at the electrode surface. Based on this concept, we explain why adding a thin semiconductor layer (1 to 10 nm) on a metal electrode can enhance electrocatalytic activity, which may guide the discovery of thin-film catalysts. We also establish that the physical limit for the exchange current density reachable for the hydrogen evolution reaction is 10 A cm^-2^ for an ideal material.

## Abstract (web summary)

Understanding the electrode-electrolyte interface is crucial for sustainable electrocatalysis. Here, the authors introduce a model including electronic equilibrium at the interface, showing that the Helmholtz potential controls activity, which can be enhanced by thin semiconductor layers.

## Introduction

Chemical energy carriers such as hydrogen will play a central role in the transition to a more sustainable society. However, for green hydrogen technologies to become economically viable, it is crucial to develop cost-effective and highly active catalysts for water splitting. Significant progress has been made in replacing noble metals with earth-abundant elements for the oxygen evolution reaction (OER), particularly with the development of transition metal hydroxide catalysts. In contrast, the hydrogen evolution reaction (HER), the hydrogen oxidation reaction (HOR), and the oxygen reduction reaction (ORR)—all critical for electrolyzers and fuel cells—still heavily rely on noble metals like Pt and Ir^1^.

A pivotal breakthrough came in 2011, when Markovic and co-workers discovered that decorating Pt surfaces with Ni(OH)₂ nanoclusters significantly enhanced HER activity in alkaline media^2^. Remarkably, this strategy was later shown to improve HER performance across a wide range of metals^3^. Even Au, a notoriously poor HER catalyst, could be made nearly as active as Ni, which is widely used in industrial electrolyzers. While the example of Au is not ideal for reducing our reliance on noble metals, it highlights the immense potential of interface engineering to enhance catalytic performance.

Beyond the HER, the Ni(OH)₂ decoration strategy was also successfully extended to the HOR^4^, CO oxidation reaction^5^, and, more recently, to the ORR^6^. Initially, the enhanced HER activity was attributed to synergistic effects at the Pt–Ni(OH)₂ interface. Specifically, Ni(OH)₂ was believed to provide adsorption sites for OH⁻ ions, which would otherwise poison the Pt surface, thereby promoting the water dissociation step (Volmer step). This atomistic hypothesis was supported by DFT calculations, which demonstrated a lower activation energy for water dissociation at Pt–Ni(OH)₂ edges^7^. Hydrogen underpotential deposition measurements by Koper and co-workers further supported this view, showing that the hydrogen adsorption barrier had to be influenced by the presence of Ni(OH)₂, since the hydrogen adsorption energy remained unchanged between bare Pt(111) and Ni(OH)₂-decorated Pt(111)^8^. For the HOR, CO oxidation reaction, and ORR, similar principles apply. In the case of the HOR, for example, Ni(OH)₂ nanoclusters are believed to act as reservoirs for OH⁻, enhancing reaction kinetics.

However, subsequent studies challenged the original explanation by Markovic and co-workers. Koper and co-workers showed that HER activity did not scale with the perimeter of Ni(OH)₂ nanoclusters, as would be expected if only the Pt–Ni(OH)₂ edges were catalytically active^9^. Instead, they adopted a more surface science-oriented approach. According to their revised model of the pH-dependence of the HER—based on the energy penalty associated with charge transfer across the 2D interfacial water network^8^—they showed that the presence of Ni(OH)₂ nanoclusters shifts the potential of maximum entropy (pme), i.e., the potential at which the interfacial water network is the weakest, towards more negative potentials. As the pme gets closer to the onset of the HER (like in acidic electrolytes), charge transfer across the interfacial water network is facilitated.

The origin of the pme shift induced by Ni(OH)₂ nanoclusters, however, is nontrivial. Koper and co-workers referred to earlier work by Feliu and co-workers, which investigated water reorientation at Pt(111) surfaces modified with various adatoms^10^. In that study, the evolution of the pme in the presence of adatoms was attributed to the formation of a surface dipole caused by partial electron transfer between the less electronegative atom and the more electronegative one. For instance, Pb adatoms—being less electronegative than Pt—partially transfer charge to Pt, creating a positive surface charge. This positive surface charge modifies the surface’s electron affinity and thereby shifts the pme towards lower potentials, meaning a lower potential is required to reorient the water molecules into a configuration favorable for the HER. A quantitative physical formalism of these interfacial processes that could be applied to more complex catalytic systems is, however, still lacking.

Here, we propose a global model grounded in solid-state physics to describe the metal/semiconductor/electrolyte interface in the context of electrocatalysis. Specifically, we introduce the interfacial potential barriers and electric fields induced by charge transfer into the Butler-Volmer equation. This approach allows us to unify both the atomistic and surface science-based models proposed in the literature. Notably, it elucidates the linear relationship between the overpotential and the work function (WF) of metal electrodes, particularly in the context of the HER, which was observed for the first time by Trasatti in 1972^11,12^. Our interfacial approach, including local effects of the electrolyte structure, extends the recent formalism proposed by Buckley and Leddy, relating the Galvani potential to the Gibbs free energy of the reaction^13^. The model’s predictions are in excellent agreement with experimental data from the literature, encompassing both bare metal and hydroxide-modified surfaces for the HER in both acidic and alkaline media. Based on this global model, we predict the dependence of the electrode material’s electrocatalytic activity^14^ with its electronic properties, allowing us to propose rational design strategies for electrocatalysts.

## Results and discussion

### Metal electrodes

#### Butler-Volmer formalism

In an electrochemical cell, when an overpotential $\eta$ is applied to a metallic working electrode with respect to the equilibrium potential ${E}_{{eq}}$, a current flows due to the electrochemical reaction. For a unimolecular redox reaction, ${ox}+z{e}^{-}\to {red}$, Butler and Volmer proposed that the current varies exponentially with the applied overpotential to explain Tafel’s observations a few decades earlier. Bridging the gap between thermodynamics and reaction kinetics for electrochemical systems, the extended Butler-Volmer equation^15^ describes the current density $j$ combining two opposite terms accounting for the reduction (right-hand term) and the oxidation (left-hand term) reactions, as follows:

1 $$j={j}_{0}\left(\frac{{[{red}]}_{{surf}}}{{[{red}]}_{{bulk}}}e^{\frac{\left(1-\alpha \right){zF}\eta }{{RT}}}-\frac{{[{ox}]}_{{surf}}}{{[{ox}]}_{{bulk}}}{e}^{-\frac{\alpha {zF}\eta }{{RT}}}\right),$$

where ${j}_{0}$ is the pre-exponential exchange current density, $z$ is the number of electrons involved in the reaction, *F* is the Faraday constant, *R* is the universal gas constant, *T* is the temperature in Kelvin, *α* is the charge transfer coefficient, and ${[{red}/{ox}]}_{{surf}/{bulk}}$ the surface and bulk concentrations of the redox species. The charge transfer coefficient $\alpha$ describes the symmetry of the reaction in terms of how easily electrons are transferred one way or the other, and should have a value close to 0.5, as determined by the model of energy fluctuations^15,16^. This well-established description is still in use today. Often, the bulk and surface concentrations are considered identical. But for large overpotentials, the exponential behavior is limited by charge diffusion in the electrolyte, as observed for the ORR, for instance. A more refined description would involve accounting for mass transport to determine the surface concentration^15^ as well as the concentration of reactants adsorbed at the surface, as discussed in the Methods 1. However, such considerations do not affect the general concept presented here, and for simplicity, we consider only the surface concentration to be relevant.

When comparing the electrocatalytic activities of various electrodes for given reactions, the differences are encompassed in the pre-exponential exchange current density ${j}_{0}$, which can be described as a function of the reactants’ concentration ($[{red}]$ and $[{ox}]$) and a kinetic reaction rate ${k}_{0}$^15^:

2 $${j}_{0}=q{k}_{0}{[{red}]}_{{bulk}}^{\alpha }{[{ox}]}_{{bulk}}^{1-\alpha }.$$

${k}_{0}$ is usually further discussed through the Sabatier principle, which states that for a catalyst to be effective, it must bind reactants neither too strongly nor too weakly. In other words, one must minimize the activation free energy of the limiting step to increase the overall reaction rate. The Sabatier principle is often visualized as a volcano-shaped curve when plotting the catalytic activity vs the binding energy of a key intermediate (e.g., H* in the case of the HER), a plot originally introduced by Trasatti^11^. Most research efforts over the last few decades were directed towards the optimization of surfaces according to the Sabatier principle.

However, recent studies on metal electrodes have shown large differences in HER overpotential, which could not be described by the Sabatier principle^17^, pointing out the limits of this traditional description. In particular, the HER overpotential appears to vary as a linear function of the metal’s WF^11^. These differences are usually explained by variations of the metals’ catalytic properties or by the local structure of the interfacial water network, which are not directly described by the Butler-Volmer equation.

Recent extensions to the Butler–Volmer equation have been proposed by Leddy and co-workers to rationalize the observed correlation between the exchange current density and the metal’s WF. In their approach, this proportionality is introduced either by invoking a limiting transition state in which the electron is shared between the metal and the adsorbed hydrogen intermediate^18^, or by incorporating an activation energy term proportional to the chemical potential of the electron in the metal, drawing analogies to the Galvani potential^13^. While these models offer valuable perspectives, they rely respectively on a transition state assumption not grounded in the Sabatier principle, or on the Galvani potential, whose relevance to single metal–electrolyte interfaces is unclear.

Here, we propose a general extension of the Butler–Volmer formalism rooted in a physical description of the electrode–electrolyte interface, and more specifically in the properties of the Helmholtz layer. Within this framework, the role of the metal’s WF arises naturally and provides a rigorous justification for the energy barrier at the interface that directly influences the local reactants’ concentrations at the surface of the electrode.

#### Beyond the Sabatier principle: importance of the Helmholz layer

**Fig. 1:**
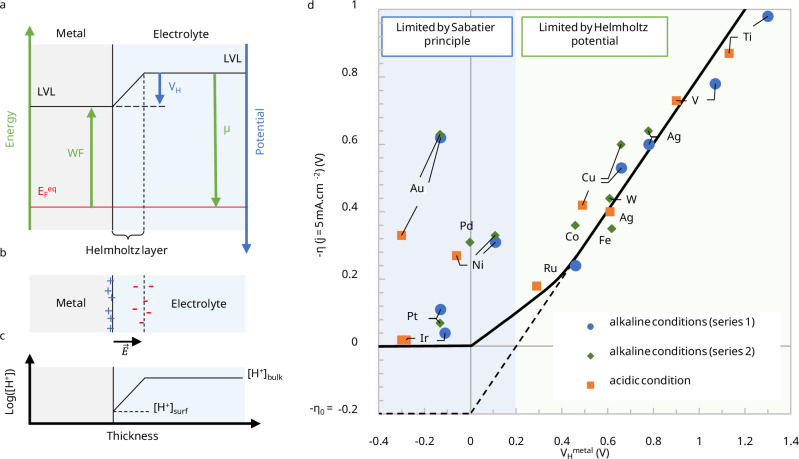
Relationship between HER overpotential (*η*)and Helmholtz potential (*V*_*H*_) for metal electrodes. **a** Energy levels in the electrode and chemical potential in the electrolyte vs the local vacuum level (LVL) and **b** charge separation at metal/electrolyte interfaces at equilibrium. All values of work function (WF) and chemical potential ($\mu$) are taken positive with the energy scale oriented upwards (green), and the potential scale oriented downwards (blue). For ${WF}$ smaller than $-\mu$, holes accumulate at the electrode surface while counter anions accumulate in the electrolyte, building the double layer until the Helmholtz potential compensates for the initial difference between ${WF}$ and $-\mu$. For large ionic concentrations (>0.1 M), the diffuse layer can be confounded with the Helmholtz layer. In the scenario presented here, this potential barrier reduces the local proton concentration at the electrode surface (**c**), while the electrical field increases the rigidity of the water molecule network, hindering the HER. For ${WF}$ larger than $-\mu$, the charge transfer and potential variation are opposite, and the HER is not limited by the interface potential. **d** Comparison between the HER overpotential $-\eta$ measured at −5 mA cm^−2^ and ${V}_{H}=-(\mu+{WF})/q$ for different metals in both acid (0.1 M HClO_4_) and alkaline conditions (0.1 M KOH), respectively, taken from ref. ^3^ (series 1) and^20^ (series 2). The chemical potential $\mu$ has been calculated using the Nernst equation to −5.2 eV and −5.0 eV vs LVL, considering a local surface pH of 13 and 10 for the alkaline and acid conditions, respectively. When the Helmholtz potential limits the reaction (green area), all 15 measurements align with the theoretical model (black solid line), considering η_0_ = 0.2 V. For values of ${V}_{H}$ close to zero or negative, the Helmholtz potential is not limiting the reaction anymore, and surface catalytic properties dominate. WF values were taken from ref. ^28^. They are summarized in Supplementary Table 1. Source data are provided as a Source Data file.

When a metal electrode is put into contact with an electrolyte, the potential energy difference between the electrons inside the metal, defined as the Fermi level ${E}_{F}$ and set by the metal’s ${WF}$, and the electrons in solution, defined by the chemical potential $\mu$, leads to a charge transfer until equilibrium is reached (Fig. 1a, b). Usage defines the ${WF}$ positive (e.g., $W{F}_{{Pt}}\simeq 5.3\,{\mathrm{eV}}$) and $\mu$ negative (e.g., ${\mu }^{{SHE}}=-4.44\,{\mathrm{eV}}$) as oriented in Fig. 1a. When equilibrium is reached, the potential variation in the double layer compensates exactly the initial potential difference, and no net current is observed.

The Helmholtz layer, composed of adsorbed ions on the metal surface, is only a few angstroms thick, while the Gouy-Chapman layer, composed of an ion concentration gradient in the electrolyte, has a typical thickness defined by the Debye length, which depends on the ionic strength of the solution^16^. For concentrated electrolytes (> 0.1 M), the Debye length is only about 1 nm thick, so that the double layer can be modeled by a simple linear Helmholtz potential drop ${V}_{H}$ depending on the ${\mathrm{WF}}$ of the metal electrode and the chemical potential of the electrolyte $\mu$ (Fig. 1a)^16^:

3 $${V}_{H}^{{metal}}=-\frac{\mu+{WF}}{q}.$$

The Helmholtz potential ${V}_{H}$ leads to a potential barrier for ions to reach the surface. In other words, the electric field building up near the electrode prevents the displacement of charged reactants or products from/to the surface. This purely electrostatic effect takes place in addition to catalytic effects and the Sabatier principle that considers the binding energy and how the reaction occurs at the molecular level. In order to facilitate charge transfer, the Helmholtz potential at the electrode surface must be minimized, meaning that the ${WF}$ of the surface should be as close as possible to the chemical potential of the electrolyte. In this case, the electric field at the electrode surface is minimized and has limited impact on the water dipoles, maximizing the electrolyte’s entropy. The interfacial water network is less “rigid”, minimizing the reorganization energy. This potential corresponds to the pme, which may slightly vary from the point of zero charge (PZC) depending on the chemical interaction between the water molecules and the catalyst surface^19^. This explains the observation of Feliu and co-workers^10^ when adding Pb atoms on a Pt(111) surface, which lowers the ${\mathrm{WF}}$ of the surface closer to the chemical potential of the electrolyte.

The Helmholtz potential provides a physical basis for the energy barrier introduced by Buckley and Leddy^13^. On a range of different metal electrodes, their description demonstrates a linear relationship between $\log ({j}_{0})$ and the ${\mathrm{WF}}$, for the HER as well as for the reaction ${{{\rm{Fe}}}}^{3+}+{e}^{-}={{{\rm{Fe}}}}^{2+}$, showing the general relevance of the ${\mathrm{WF}}$ for electrochemistry. Our electronic description based on the electrochemical equilibrium at the solid-liquid interface reaches similar conclusions for the HER on metals from another perspective, as described in subsection 2.3. In the case of HER, the great advantage of our model, however, is that it encompasses the interfacial behavior of the electrolyte while being quantitative. It is particularly adapted to more complex heterostructures such as semiconductor thin films deposited on metal supports (section 3), which are not easily described by previous extensions of the Butler-Volmer formalism.

#### Application to the hydrogen evolution reaction

The essential role of the minimization of the Helmholtz potential is demonstrated with the HER, which is a two-electron reaction not limited by the diffusion of species in the electrolyte. The extended research on HER allows a quantitative analysis of this model based on existing literature. First, let us consider the reduction reaction of protons in acidic media:

4 $${{{\rm{H}}}}_{\left({aq}\right)}^{+}+{e}^{-}=\frac{1}{2}{{{\rm{H}}}}_{2(g)}.$$

The local concentration of the ion at the surface of the electrode is described by the ratio of ${\left[{{{\rm{H}}}}^{+}\right]}_{{surf}}$ with ${\left[{{{\rm{H}}}}^{+}\right]}_{{bulk}}$ in Eq. (1). As the energy needed for the ion to reach the surface across the Helmholtz layer is $q{V}_{H}$, using the Boltzmann distribution, we can derive:

5 $$\frac{{\left[{{{\rm{H}}}}^{+}\right]}_{{surf}}}{{\left[{{{\rm{H}}}}^{+}\right]}_{{bulk}}}={e}^{-\frac{q{V}_{H}}{{k}_{B}T}}={e}^{-\frac{F{V}_{H}}{{RT}}}.$$

The surface concentration of the proton is strongly decreased as represented in Fig. 1c, even for small Helmholtz potentials, as ${{{\rm{k}}}}_{{{\rm{B}}}}{{\rm{T}}}\sim 25{{\rm{meV}}}$ only at room temperature. For the HER to occur, the interfacial potential drop must be compensated by applying an overpotential that we call the Helmholtz overpotential ${\eta }_{H}$. For diffusion-limited reactions, the “bulk” concentration considered here corresponds to the concentration at the edge of the Helmholtz layer near the surface, which can be determined using mass transport models^15^ as explained in Methods 1, with the same formalism as that developed in Methods 2.

By considering this local concentration in the expression of the cathodic current Eqs. (1, 5), we obtain:

6 $$j=-{j}_{0}{e}^{-\frac{\alpha {zF}}{{RT}}\left(\eta+{\eta }_{H}\right)}\,{{\rm{with}}}\,{\eta }_{H}=\frac{{V}_{H}}{z\alpha }.$$

In this expression, the limiting exchange current density, ${j}_{0}$, is independent of the Helmholtz potential, i.e., independent of the metal’s electronic properties, but can still be qualitatively interpreted within the framework of the Sabatier principle, although it is not explicitly included in the extended Butler–Volmer equation Eq. (1). ${j}_{0}$ is therefore the highest exchange current density possible for the HER, reached for an idealized proton diffusion at the interface and neglecting the anodic reverse reaction. It can be determined experimentally for an overpotential canceling the Helmholtz overpotential, i.e., $\eta=-{\eta }_{H}$.

When comparing electrode materials, the overpotential $\eta$ is commonly reported at a given current density (typically at $j=$−5 mA/cm^−2^). Considering the logarithm of the Eq. (6), one can find that

7 $$\eta+{\eta }_{H}=\frac{{RT}}{\alpha {zF}}{{\mathrm{ln}}}\left(-\frac{{j}_{0}}{j}\right)={\eta }_{0}.$$

${\eta }_{0}$ is a constant of the electrochemical reaction and only reflects the intrinsic properties of the surface, such as the density of charge carriers or its catalytic properties, independently of any electrostatic effects. The overpotential $\eta$ depends on the Helmholtz overpotential and thus on the difference between the metal’s WF and the chemical potential of the electrolyte (Eq. 3).

In the case of the HER, where $z=2$ and $\alpha \sim 0.5$, the negative overpotential is commonly reported as its positive absolute value. It corresponds here to $-\eta$, and thus appears directly proportional to the Helmholtz potential:

8 $$-\eta={V}_{H}-{\eta }_{0},$$

as validated based on existing data across three independent sets of experiments, as shown in Fig. 1d, both in alkaline and acidic conditions^3,20^.

Between the alkaline and acidic conditions, the chemical potential of the solution is corrected for the local pH close to the surface (at a few Debye lengths from the electrode surface) as discussed in the Methods 1. Under alkaline conditions (0.1 M KOH), the local pH remains close to the bulk value of pH = 13. However, under acidic conditions (0.1 M HClO_4_), the experimental measurements fit the model considering a local pH ≈ 10. This local alkaline environment is due to the consumption of H^+^ during the reaction. Such strong pH swings during acidic HER have been reported for Pt before, especially in the presence of ClO_4_^-^ ions, reaching values up to pH = 12^21^.

The linear behavior is respected for all metals with WFs below 4.7 eV (Ru, Co, Fe, Ag, W, Cu, V, Ti), with ${\eta }_{0}$ = 0.2 V. Interestingly, this value allows us to estimate ${j}_{0}$ for the HER, which is fitted to ${j}_{0}=10$ ${\mbox{A.c}}{{\mbox{m}}}^{-2}$. It can be interpreted as the physical limit for the exchange current density for the HER, under the considered experimental conditions and for the metals studied, assuming ideal diffusion of protons to the electrode surface and no reverse reactions. Pushing beyond this limit would require considering other types of catalysts or non-ambient conditions.

For overpotentials approaching zero, the anodic contribution in the Butler-Volmer equation can no longer be neglected in Eq. (7). The corresponding theoretical curve (solid line in Fig. 1d), which accounts for both anodic and cathodic currents, is calculated as detailed in the Methods 2. In this model, the surface and bulk H_2_ concentrations are set to their saturation values. At large Helmholtz potentials, the response remains linear, as previously discussed. However, when ${V}_{H}^{{metal}}=0.43\,{{\mbox{V}}}$ under our conditions, the reverse reaction becomes significant, requiring a higher overpotential than predicted by the linear approximation (dashed line) to sustain the net current. For negative Helmholtz potentials, the current is instead limited by proton diffusion toward the electrode surface.

For metals such as Ni, Pt, Pd, Au, and Ir, whose WF is close to or larger than the chemical potential, only the catalytic properties of the surface drive the efficiency of the reaction and the overpotential. For instance, Pt and Ir are particularly active towards the HER because of their large WF and good catalytic properties. On the other hand, Au exhibits a high overpotential despite its large WF, because of its poor catalytic properties^22^.

### Thin film semiconductor electrodes

#### Potential variation in a semiconductor

Unlike metal electrodes, where the interfacial potential drop arises predominantly in the electrolyte through the formation of the double layer, the potential variation occurs mainly within the electrode for semiconductors. In semiconductors, charge carriers are not free, and their density is limited (below 10^17^ cm^−^^3^), so they cannot accumulate at the surface. Instead, they form a space-charge region (SCR) over a thickness ${W}_{{SC}}$ depending on the characteristics of the semiconductor such as the density of charge carriers and the potential drop (see Methods 3, Eq. (25)). Considering the continuity of the electric displacement at the interface, the Helmholtz potential is found to be negligible (see the Methods 3, Eq. (26)). The internal surface band bending reduces the Helmholtz potential in the electrolyte and shifts the locus of charge separation into the solid phase. However, the electrocatalytic activity of semiconductor electrodes is intrinsically limited by the low density of charge carriers, which constrains not only their bulk conductivity, but also the interfacial charge transfer kinetics. At the interface, the system behaves analogously to a Schottky diode, where the exchange current is directly governed by the availability of charge carriers for interfacial charge transfer processes^16^.

In practice, the charge carrier density can be increased by defect engineering or doping. But for highly doped semiconductors (> 10^19–21^ cm^−3^), the large number of charge carriers ${N}_{D}$ leads to the reduction of ${W}_{{SC}}\propto {N}_{D}^{-1/2}$ (see Eq. (25)), so that ${V}_{H}$ is no longer negligible. Ultimately, the number of charge carriers increases until the SCR can be considered as surface charge, like in a metal electrode. In this case, ${V}_{H}$ behaves as previously described and can only be reduced by sufficiently high WFs (set by the energy level of the dopant). This is the case for boron-doped diamond (${N}_{D}\sim$ 10^21^ cm^−3^), which has a WF = 5.1–5.3 eV and shows no potential drop in the electrolyte^23^. Yet, the C–H surface bonds of diamond have a considerable adsorption energy (up to 3 eV, compared to approximately 0 eV for Pt), which prevents efficient hydrogen evolution as the reaction intermediates remain adsorbed on the surface of the electrode^24^. Nevertheless, this explains the wide potential window of boron-doped diamond electrodes, which makes them ideal support electrodes for electrochemical studies. This stresses again that the considerations of the Helmholtz potential do not invalidate the Sabatier principle.

#### Potential variation in a thin-film semiconductor on a metal electrode

**Fig. 2:**
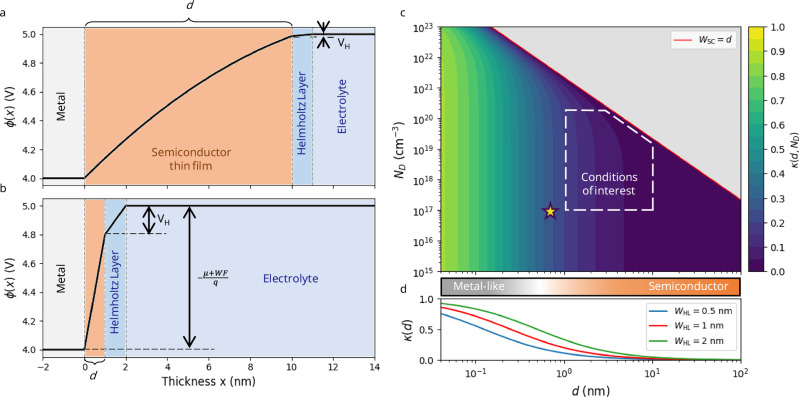
Interfacial equilibrium for semiconductor thin films on a metal electrode. Representation of the electric potential variation ($\phi$) across the electrode surface, considering a metal substrate with a work function ${WF}=4{{\rm{eV}}}$, a semiconductor with a charge carrier density *N*_*D*_ = 10^19^ cm^−3^ and relative permittivity ${\epsilon }_{r}^{{SC}}\simeq 20$, and an electrolyte with a chemical potential $\mu=-5{{\rm{eV}}}$ vs LVL, Helmholtz layer width *W*_*HL*_ = 1 nm and relative permittivity ${\epsilon }_{r}^{{HL}}\simeq 80$ for a thickness of **a** *d* = 10 nm and **b** *d* = 1 nm. Within the thin layer, the evolution of the potential is mainly influenced by the charge carrier density, while the Helmholtz potential is determined through the continuity of the electric displacement at the interface. **c** Color map of the Helmholtz potential reduction coefficient $\kappa$ as a function of the thin film thickness $d$ and the charge carrier density ${N}_{D}$ for an initial Helmholtz potential of the bare metal surface ${V}_{H}^{{metal}}=-1$ V. **d** Evolution of $\kappa$ with $d$ considering a density of charge carriers ${N}_{D}\,$= 10^17 ^cm^−3^ and different values of the Helmholtz layer width ${W}_{{HL}}$. For almost all charge carrier densities, the Helmholtz potential is strongly reduced for thicknesses between 1 and 10 nm. Optimally, the thin film should have a large doping concentration (${N}_{D}\sim$10^17–21^ cm^−3^) and a thickness of 1–10 nm to improve conductivity while minimizing $\kappa$. For $d$ larger than the width of the space charge region (${W}_{{SC}}$), the model is no longer valid. The star corresponds to the expected conditions obtained by Markovic and co-workers in ref. ^3^ and is discussed later. Source data are provided as a Source Data file.

**Fig. 3:**
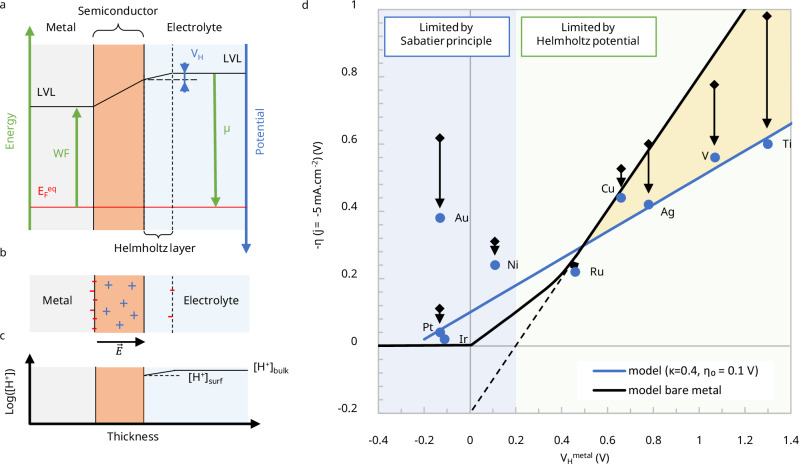
Helmholtz potential reduction at the metal/thin film semiconductor/electrolyte interface. **a**–**c** Schematic representation of charge separation at metal/semiconductor/electrolyte interfaces under equilibrium conditions. All values of work function (*WF*) and chemical potential ($\mu$) are taken positive with the energy scale oriented upwards (green), and the potential scale oriented downwards (blue). Upon contact between the semiconductor, metal, and electrolyte, charge transfer occurs until thermodynamic equilibrium is established. Unlike metals, where free charges accumulate at the surface, semiconductors possess a limited density of charge carriers. This results in the formation of a space-charge region (SCR), where charge accumulation within the bulk of the semiconductor governs the potential variation, thereby reducing the potential drop across the Helmholtz double layer. **d** Experimental observation of the Helmholtz potential reduction by a thin-film semiconductor. Comparison between the measured HER overpotential $\eta$ and the potential variation ${V}_{H}^{{metal}}=-(\mu+{WF})/q$ for different M/Ni(OH)_2_ composite electrodes. $\mu$ has been calculated to −5.2 eV considering a local surface pH of 13, accounting for the alkaline conditions. All 8 measurements, except for Au, align with the theoretical model (blue solid line) considering $\kappa=0.4$ and η_0_ = 0.1 V. The black solid line represents the bare metal electrode behavior. The difference in ${\eta }_{0}$ indicates that the M/Ni(OH)_2_ composites are less efficient than the bare metals, likely due to fewer accessible active sites. In the region of positive Helmholtz potential, the improvement in the overpotential from the bare metal surfaces (presented in Fig. 1) is represented by black arrows. Overpotential values (measured at −5 mA cm^−2^ in 0.1 M KOH) were taken from ref. ^3^. *WF* values were taken from ref. ^28^. They are summarized in Supplementary Table 2. Source data are provided as a Source Data file.

The accumulation of charge in an *n*-type semiconductor thin film atop a metal electrode can be leveraged to effectively reduce the Helmholtz potential (Figs. 2a, b, 3a). The potential at the metal-semiconductor interface is given by the ${WF}$ of the metal, while the potential at the semiconductor-electrolyte interface is given by $-\mu /q-{V}_{H}$. The charge transfer upon contact leads to the formation of an SCR in the semiconductor that diminishes the potential drop across the Helmholtz layer (Fig. 3b).

The evolution of the potential $\phi (x)$ in the semiconductor (Fig. 2a, b) can be derived from the Poisson equation with a uniform charge density $q{N}_{D}$ as described in the Methods 4. The Helmholtz potential ${V}_{H}^{{SC}}$ at the semiconductor surface can be derived from Eq. (3):

9 $${V}_{H}^{{SC}}=\kappa {V}_{H}^{{metal}},$$

with

10 $$\kappa=\frac{1-\frac{{d}^{2}}{{W}_{{SC}}^{2}}}{1+\frac{{\epsilon }_{r}^{{HL}}}{{\epsilon }_{r}^{{SC}}}\frac{d}{{W}_{{HL}}}}{\to }_{d\ll {W}_{{SC}}}\frac{1}{1+\frac{{\epsilon }_{r}^{{HL}}}{{\epsilon }_{r}^{{SC}}}\frac{d}{{W}_{{HL}}}},$$

${V}_{H}^{{metal}}$ being the Helmholtz potential at the metal surface, ${W}_{{HL}}$ the thickness of the Helmholtz layer, ${\epsilon }_{r}^{{SC}}$ and ${\epsilon }_{r}^{{HL}}$ the relative permittivity of the semiconductor and the Helmholtz layer, respectively, ${N}_{D}$ the density of charge carriers in the semiconductor, and ${W}_{{SC}}$ the width of the SCR that would accommodate the potential drop at the semiconductor-metal interface:

11 $${W}_{{SC}}=\sqrt{\frac{2{\epsilon }_{0}{\epsilon }_{r}^{{SC}}}{q{N}_{D}}{V}_{H}^{{metal}}}.$$

In practice, ${W}_{{HL}}$ can be estimated by the Debye length in the electrolyte depending on the ion concentration (typically 1 nm at 0.1 M). The relative permittivity of the electrolyte ${\epsilon }_{r}^{{HL}}$ usually remains close to that of bulk water, so that ${\epsilon }_{r}^{{HL}}\simeq 80$, although it is known to decrease slightly with increasing ionic strength ^25^. The relative permittivity of the thin layer ${\epsilon }_{r}^{{SC}}$ is material-dependent.

In the case of semiconductor thin films with a thickness $d < {W}_{{SC}}$, the entire film becomes charged and accommodates some of the potential variation. When $d\sim {W}_{{SC}}$, the film is thick enough to cancel out the Helmholtz potential completely and $\kappa \sim 0$ (Fig. 2a). When $d < < {W}_{{SC}}$, the semiconductor layer is insufficient to fully compensate for the potential drop at the interface (Fig. 2b). When $d > {W}_{{SC}}$, the two interfaces of the semiconductor layer begin to decouple, forming two separate SCRs on the metal and on the electrolyte side. In this case, the electrode behaves mostly like a bulk semiconductor electrode.

While the theoretically optimal film thickness to fully cancel the Helmholtz potential is $d={W}_{{SC}}$, achieving this condition in practice is challenging, as ${W}_{{SC}}$ depends on both the semiconductor dielectric constant ${\epsilon }_{r}^{{SC}}$ and the charge carrier density ${N}_{D}$, which are often difficult to determine accurately and to control experimentally. Moreover, increasing the film thickness to match ${W}_{{SC}}$ in the low-doping regime can introduce additional limitations to charge transport. In the limit ${N}_{D}\to 0$, the activity of the electrode is limited by the resistivity of the film and the low interface charge carrier density, enabling the reaction (see the Methods 5). Importantly, a strong reduction of the Helmholtz potential exceeding 90% can already be achieved with thin layers satisfying $d\ll {W}_{{SC}}$, as shown in Fig. 2c.

For practical use, any reasonable value of charge carrier density (${{{\rm{N}}}}_{{{\rm{D}}}}\sim$10^17–21^ cm^−3^) and thickness in the range of 1–10 nm will greatly decrease the effect of the Helmholtz potential with $\kappa < 0.2$ (Fig. 2d). However, maximizing the charge carrier density can increase charge transfer at the interface and the reaction rate, as exemplified later. The control of the doping and thickness within the suggested range can be achieved using various techniques (e.g., electrochemical deposition, pulsed laser deposition, chemical vapor deposition, atomic layer deposition, ion beam sputtering, etc.) through traditional substitutional doping but also through native defects (e.g., oxygen vacancies, interstitial atoms) as it is the case in the experimental results presented here (see Supplementary Note 1).

#### Application to the hydrogen evolution reaction

The linear dependency of ${V}_{H}^{{SC}}$ as a function of ${V}_{H}^{{metal}}$ was reported in the experimental work by Markovic and co-workers, comparing the overpotential of various metals covered with Ni(OH)_2_ under the same conditions (Fig. 3d)^3^. A value of $\kappa=0.4$ can be extracted from these experimental data by considering the slope of the corresponding linear regression. As shown previously (Eq. (8)), the overpotential $-\eta$ scales linearly with the Helmholtz potential of the system, which in the present case corresponds to ${V}_{H}^{{SC}}$. Using the definition of $\kappa$ introduced in Eq. (9), this relationship can be rewritten as $-\eta=\kappa {V}_{H}^{{metal}}-{\eta }_{0}$. The value of *κ* can be derived theoretically from Eq. (10) considering $d\ll {W}_{{SC}}.$ The thickness of the Helmholtz layer is estimated to be on the order of 1 nm, consistent with the Debye length expected for electrolytes at a concentration of 0.1 M under the experimental conditions considered here. At these concentrations, the relative permittivity of the electrolyte remains close to that of bulk water (${\epsilon }_{r}^{{HL}}\simeq 80$)^25^. Determining the permittivity of the Ni(OH)_2_ thin film is more complex. A pure nickel hydroxide electrode can have a permittivity as low as 4, which increases up to several tens because of oxidation and water absorption, for example, after cycling in an alkaline cell^26,27^. In practice, the porous nature of the clusters and their partial infiltration by the electrolyte further increase the relative permittivity of the thin layer. Assuming a thickness $d=0.7$ nm for the Ni(OH)_2_ clusters on Pt(111), as reported from STM measurements^2^, and the value $\kappa \simeq 0.4$, the corresponding permittivity of the Ni(OH)_2_ thin film is estimated to be ${\epsilon }_{r}^{{SC}}\simeq 37$. Furthermore, for sub-nanometric layers, other factors such as electron transfer through tunneling should also be considered, which would lead to a behavior closer to that of the bare metal electrode ($\kappa=1$), thereby increasing the experimentally observed value of $\kappa$ and the estimated value of ${\epsilon }_{r}^{{SC}}$.

Notably, ${\eta }_{0}$ = -0.1 V is more negative compared to that of the bare metal electrodes due to two main factors (Fig. 3d). First, the catalytic properties of the surface and charge density of the electrode covered with Ni(OH)_2_ are generally less favorable for the reaction than those of the bare metal, resulting in a larger overpotential. Second, only a portion of the electrode surface is covered with Ni(OH)_2_, which reduces the effective active area. As a result, the local current density on the active Ni(OH)_2_-covered regions must be higher to reach the same overall current density of 5 mA cm^−2^ (based on the total geometric surface area). The thin semiconductor layer is therefore mostly of interest for large ${V}_{H}^{{metal}}$. Additionally, catalytic factors such as the need for exposed platinum sites for reactions involving surface intermediates must also be considered. However, the idea that Ni(OH)_2_ islands necessarily reduce the electrochemically active surface area of the electrode remains under debate. Koper *et al*. recently provided initial experimental evidence^9^, based on CO stripping voltammetry and infrared spectroscopy, suggesting a possible direct contribution of Ni(OH)_2_ to the HER. This interpretation is consistent with our model, which predicts a deep depletion layer in such thin Ni(OH)_2_ films and, consequently, a high density of charge carriers available to sustain the reaction. Nevertheless, further experimental work is required to conclusively resolve this issue. In the case of Au, the reduction of the overpotential from 0.68 V to 0.38 V is likely due to the better catalytic properties of the Au/Ni(OH)_2_ composite compared to bare Au, since the Helmholtz potential is not limiting.

**Fig. 4:**
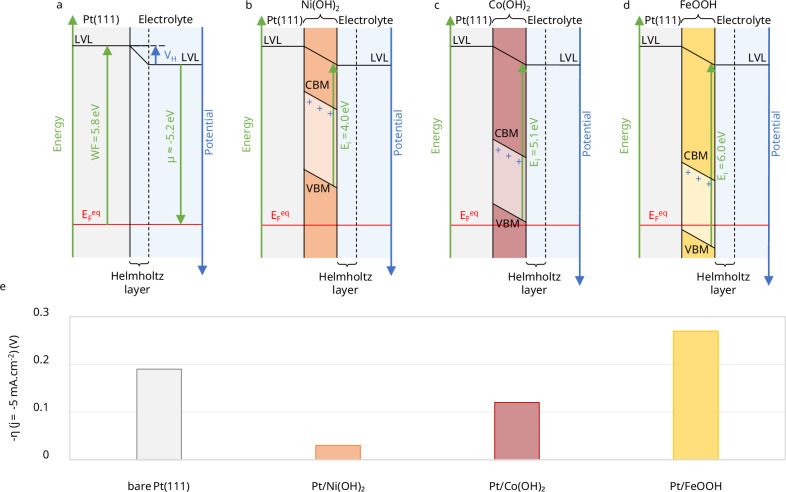
Modulation of the HER overpotential on the Pt(111) surface by a thin metal hydroxide layer. Band alignment and interfacial equilibrium for a Pt(111) surface **a** bare, covered with a thin film of **b** Ni(OH)_2_, **c** Co(OH)_2_, and **d** FeOOH. Trapped charges from *n*-type dopants are indicated by blue “+” signs. **e** The HER overpotential values of these surfaces measured at −5 mA cm^−2^ in 0.1 M KOH, taken from ref. ^5^. The WF value of Pt(111) was taken from ref. ^28^. The electronic properties of 2D Ni(OH)_2_, 2D Co(OH)_2_ and nanosized FeOOH were taken from refs. ^29–34^, respectively. The bandgap energy of 2D Co(OH)_2_ was extrapolated from the Tauc plots based on ref. ^30^. In the case of nanosized FeOOH, the γ/δ polymorphs were considered. All values are summarized in Supplementary Table 3. Source data are provided as a Source Data file.

Ni(OH)_2_ has been reported to exhibit higher catalytic activity on Pt(111) surfaces compared to other semiconductors such as Co(OH)_2_ and FeOOH^5^. This difference can be explained by variations in their ionization energies (${E}_{i}$), which influence the charge transfer processes at the interface. The high ${\mathrm{WF}}$ of Pt(111) results in a negative Helmholtz potential when in contact with an aqueous electrolyte (Fig. 4a). Although this negative Helmholtz potential does not reduce the proton concentration at the electrode surface, it introduces an energetic penalty associated with charge transfer across the two-dimensional interfacial water network, as previously described by Koper and co-workers^8^, leading to an overpotential of 0.19 V^5^.

Depositing a thin semiconductor layer can mitigate the Helmholtz potential, provided the Fermi level of the system drops below the *n*-type dopant states, enabling the accumulation of positive trapped charges. This effect is observed for Ni(OH)_2_, Co(OH)_2_, and FeOOH (see Fig. 4b–d). However, the concentration of surface charge carriers can vary depending on the ionization energy of the material, which dictates the Fermi level position relative to the valence band maximum (VBM).

A high density of charge carriers is critical, as the exchange current in semiconducting materials is directly proportional to the number of available carriers at the surface. In Ni(OH)_2_ and Co(OH)_2_, the Fermi level lies below the VBM, establishing a deep depletion layer. A deep depletion condition is achieved when the ionization energy is lower than both the metal WF and the chemical potential of the electrolyte. Under these conditions, the system behaves similarly to a metal, with both mobile charges, ensuring good charge exchange, and immobile (trapped) charges contributing to the compensation of the Helmholtz potential. This leads to a notable reduction in overpotential, as illustrated in Fig. 4e.

In contrast, for FeOOH, the Fermi level remains within the bandgap, which limits the accumulation of surface charge carriers. As a result, the overpotential is even higher than that of the bare Pt surface, likely due to both less favorable catalytic properties and a reduced carrier density. As such, most of the reaction occurs on the bare Pt surface, whose active surface area is decreased by the presence of FeOOH.

#### Prospects

The electronic properties of thin-film catalysts significantly affect the electrode-electrolyte interface. In this analysis, we introduce the effect of the charge and reactant concentrations at the electrode-electrolyte interface in the Butler-Volmer formalism to explain recent experimental results from the literature regarding HER electrocatalytic activity. We show that reaction kinetics are not only influenced by the Sabatier principle, but also by the Helmholtz potential at the electrode surface, which should be minimized. Indeed, the Helmholtz potential creates a surface electric field that impacts the local concentration of reactants as well as the entropy of the electrolyte or the “rigidity” of the water hydrogen bond network. The linear dependency of the HER overpotential with the work function of metal electrodes, as well as their activity enhancement by hydroxide thin films, is explained by these findings. This global understanding of interfacial processes will facilitate the knowledge-based design of electrocatalysts for a given target reaction. The minimization of the Helmholtz surface potential can be achieved by:

1. Tuning the work function to the chemical potential of the electrolyte, for instance, ${{\rm{WF}}}\simeq 5$ eV for HER. While this approach has historically been taken with noble metals, other materials with high WF can also be used if they exhibit appropriate catalytic properties.
2. Adding a thin semiconductor layer (1 to 10 nm) atop the metal current collector, which will shift the potential variation in the electrode material, creating a solid-state double layer. This has been found empirically with Ni(OH)_2_ thin films on various metals for the HER. By choosing semiconductors with a VBM above the Fermi level of the interface (${E}_{i} < {WF}$ and ${E}_{i} < -{{\rm{\mu }}}$), the deep depletion of the layer ensures a large density of charge carriers at the interface and increases reaction kinetics.
3. Shifting the chemical potential of the electrolyte through changes in its composition.

Once the Helmholtz potential is reduced, the surface catalytic properties of the electrode can be optimized according to the Sabatier principle, by tuning the composition of the thin film, its surface termination, or depositing selected nanomaterials and single atoms.

Partial oxidation, surface reconstruction, and chemical modification under bias naturally fit within the proposed framework, as they effectively modify the electronic parameters governing the interface, such as the work function or electron affinity or even create a semiconductor thin film. This highlights the importance of *operando* characterization of surface chemistry and electronic properties, including for carbon-supported catalysts and emerging materials such as alloys and high-entropy alloys. Such an approach opens clear pathways for rational interface engineering and performance optimization through simultaneous control of the substrate, catalyst, and electrolyte.

The fundamental role of the Helmholtz potential on charge transfer efficiency extends to any electrochemical reaction in any type of electrolyte. Its presence at the interface arises systematically from charge equilibration and, in turn, governs ion distributions, interfacial electric fields, the orientation of polar intermediates, electrolyte structuring, and transport properties. Design strategies to suppress water electrolysis, as for aqueous batteries, should consider materials with low WF, such as Ti and V. Beyond the HER, these considerations apply to other significant electrochemical reactions, such as ORR or CO2RR. We anticipate that leveraging this concept would also benefit the fields of photo(electro)catalysis and colloidal nanoparticle chemistry, and that it could be further extended to non-aqueous systems for electrochemical energy storage by considering their respective chemical potentials.

## Methods

### Diffusion limited reactions and adsorption

The mechanisms of reactions on surfaces and heterogeneous catalysis are driven by the adsorption of reactants on the surface and the desorption of products from the surface. In the case of the HER, two different reaction schemes can be derived in the case of acidic (12) or alkaline conditions (13):

12 $${{{\rm{H}}}}^{+}+{e}^{-}=\frac{1}{2}{{{\rm{H}}}}_{2}$$

13 $${{{\rm{H}}}}_{2}{{\rm{O}}}+{e}^{-}=\frac{1}{2}{{{\rm{H}}}}_{2}+{{\rm{O}}}{{{\rm{H}}}}^{-}$$

Considering the simple competing mechanism of the Langmuir adsorption, we can derive the fraction of the reactant adsorbed at the surface $\theta$ as a function of the concentration of the competing species at the surface of the electrode and the equilibrium constants $K$. In the case of acidic conditions, protons, dihydrogen molecules, and water molecules can adsorb, so that:

14 $${\theta }_{{{{\rm{H}}}}^{+}}=\frac{{K}_{{{{\rm{H}}}}^{+}}{\left[{{{\rm{H}}}}^{+}\right]}_{{surf}}}{1+{K}_{{{{\rm{H}}}}_{2}{{\rm{O}}}}{\left[{{{\rm{H}}}}_{2}{{\rm{O}}}\right]}_{{surf}}+{K}_{{{{\rm{H}}}}_{2}}{\left[{{{\rm{H}}}}_{2}\right]}_{{surf}}+{K}_{{{{\rm{H}}}}^{+}}{\left[{{{\rm{H}}}}^{+}\right]}_{{surf}}}$$

However, the concentration of water molecules remains essentially constant and is much higher than that of the protons consumed in the reaction or the dihydrogen molecules, which form gas bubbles due to their low solubility. The expression can be simplified as:

15 $${\theta }_{{{{\rm{H}}}}^{+}}\sim \frac{{K}_{{{{\rm{H}}}}^{+}}{\left[{{{\rm{H}}}}^{+}\right]}_{{surf}}}{{K}_{{{{\rm{H}}}}_{2}{{\rm{O}}}}{\left[{{{\rm{H}}}}_{2}{{\rm{O}}}\right]}_{{surf}}}\propto {\left[{{{\rm{H}}}}^{+}\right]}_{{surf}}$$

**Fig. 5:**
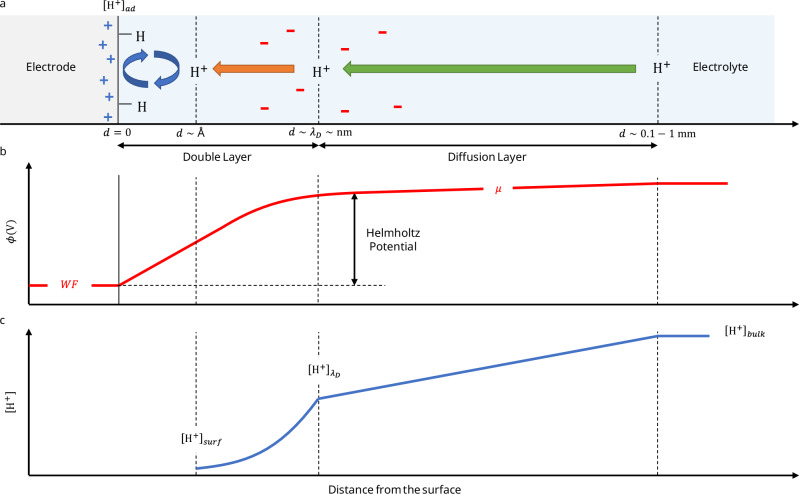
Multiscale electrochemical structure and transport at the electrode-electrolyte interface. **a**–**c** Schematic representation of charge accumulation (**a**), electrical potential profile (**b**), and proton (H^+^) concentration gradients (**c**) as a function of distance from the electrode surface. Note that the horizontal axis employs a non-linear scale to represent multiscale phenomena across the electrical double layer and the bulk diffusion layer. In the diffusion layer, the concentration profile is governed by long-range mass transport (green arrow). Within the compact and diffuse double layers, the ion distribution is modulated by the Helmholtz potential variation (orange arrow). At the immediate electrode surface, the interfacial concentration is dictated by adsorption mechanisms and electrochemical redox reactions (blue arrows).

The proportion of the catalytic sites occupied by protons is simply proportional to the concentration of protons at the surface. This concentration is determined by the gradient of the Helmholtz potential in the Helmholtz layer (see Fig. 5) as defined in the main article:

16 $$\frac{{\left[{{{\rm{H}}}}^{+}\right]}_{{surf}}}{{\left[{{{\rm{H}}}}^{+}\right]}_{{\lambda }_{D}}}={e}^{-\frac{q{V}_{H}}{{k}_{B}T}}={e}^{-\frac{F{V}_{H}}{{RT}}}$$

Here, ${\left[{{{\rm{H}}}}^{+}\right]}_{{\lambda }_{D}}$ denotes the proton concentration just beyond the Helmholtz layer, at a distance $d\sim {\lambda }_{D}$ from the surface, where ${\lambda }_{D}$ is the Debye length in the electrolyte. In the case of a reaction that is not limited by diffusion, this concentration is equal to the bulk concentration as written in Eq. (5) of the main article. However, in the case of a diffusion-limited reaction, this concentration can differ from the bulk value, and in such cases, a diffusion model within the diffusion layer (indicated by the green arrow in Fig. 5) should be considered. In practice, for the data presented in the main article, we simply account for this concentration difference as a local variation in pH, which is incorporated into the model.

In the case of alkaline conditions (13), water molecules, hydrogen molecules, and hydroxide anions can adsorb, so that:

17 $${\theta }_{{{{\rm{H}}}}_{2}{{\rm{O}}}}=\frac{{K}_{{{{\rm{H}}}}_{2}{{\rm{O}}}}{\left[{{{\rm{H}}}}_{2}{{\rm{O}}}\right]}_{{surf}}}{1+{K}_{{{{\rm{H}}}}_{2}{{\rm{O}}}}{\left[{{{\rm{H}}}}_{2}{{\rm{O}}}\right]}_{{surf}}+{K}_{{{{\rm{H}}}}_{2}}{\left[{{{\rm{H}}}}_{2}\right]}_{{surf}}+{K}_{{{\rm{O}}}{{{\rm{H}}}}^{-}}{\left[{{\rm{O}}}{{{\rm{H}}}}^{-}\right]}_{{surf}}}$$

Initially, the concentration of water molecules at the surface is much greater than the hydroxide concentration, and most of the sites are occupied by water molecules. However, while being reduced, hydroxide anions are constantly produced alongside the hydrogen molecules and compete for the adsorption sites. As a result, it shifts the equilibrium toward the adsorption of hydroxide anions, effectively reducing ${K}_{{{{\rm{H}}}}_{2}{{\rm{O}}}}$ compared to ${K}_{{{\rm{O}}}{{{\rm{H}}}}^{-}}$. Furthermore, unlike hydrogen molecules, which desorb and vaporize, hydroxide anions are soluble and accumulate at the surface. ${\left[{{\rm{O}}}{{{\rm{H}}}}^{-}\right]}_{{surf}}$ can be further enhanced by the Helmholtz potential gradient when the surface of the electrode is charged positively because of its negative charge. All these considerations led to the fraction of the adsorbed molecules to be approximated by:

18 $${\theta }_{{{{\rm{H}}}}_{2}{{\rm{O}}}}\sim \frac{{K}_{{{{\rm{H}}}}_{2}{{\rm{O}}}}{\left[{{{\rm{H}}}}_{2}{{\rm{O}}}\right]}_{{surf}}}{{K}_{{{\rm{O}}}{{{\rm{H}}}}^{-}}{\left[{{\rm{O}}}{{{\rm{H}}}}^{-}\right]}_{{surf}}}$$

Even if the surface concentration of water molecules is a constant irrespective of the pH or the Helmholtz potential, its proportion adsorbed at the surface of the catalysts is not. It becomes proportional to the inverse of the concentration of hydroxyl ions at the surface, which depends on the Helmholtz potential as:

19 $$\frac{{\left[{{\rm{O}}}{{{\rm{H}}}}^{-}\right]}_{{surf}}}{{\left[{{\rm{O}}}{{{\rm{H}}}}^{-}\right]}_{{\lambda }_{D}}}={e}^{+\frac{q{V}_{H}}{{k}_{B}T}}={e}^{+\frac{F{V}_{H}}{{RT}}}$$

Thus, the proportion of water adsorbed at the surface in alkaline conditions depends on the Helmholtz potential in the exact same way the proton did in acidic conditions:

20 $${{\left[{{{\rm{H}}}}_{2}{{\rm{O}}}\right]}_{{ad}}\propto \theta }_{{{{\rm{H}}}}_{2}{{\rm{O}}}}\sim \frac{{K}_{{{{\rm{H}}}}_{2}{{\rm{O}}}}{\left[{{{\rm{H}}}}_{2}{{\rm{O}}}\right]}_{{surf}}}{{K}_{{{\rm{O}}}{{{\rm{H}}}}^{-}}{\left[{{\rm{O}}}{{{\rm{H}}}}^{-}\right]}_{{\lambda }_{D}}}{e}^{-\frac{q{V}_{H}}{{k}_{B}T}}\propto {e}^{-\frac{q{V}_{H}}{{k}_{B}T}}$$

### HER Overpotential as a function of the Helmholtz potential

In the specific case of the HER, the extended Butler–Volmer equation (Eq. (1)) can be rewritten:

21 $$\frac{j}{{j}_{0}}={{r}_{{H}_{2}}e}^{\frac{F\eta }{{RT}}}-{r}_{H+}{e}^{-\frac{F\eta }{{RT}}},{r}_{H+}=\frac{{\left[{{{\rm{H}}}}^{+}\right]}_{{surf}}}{{\left[{{{\rm{H}}}}^{+}\right]}_{{bulk}}}={e}^{-\frac{q{V}_{H}}{{k}_{B}T}},{r}_{{H}_{2}}=\frac{{\left[{{{\rm{H}}}}_{2}\right]}_{{surf}}}{{\left[{{{\rm{H}}}}_{2}\right]}_{{bulk}}}$$

For a target current density $j=-0.005$ A/cm^2^, and an exchange current density ${j}_{0}$ = 10 A/cm^2^ deduced from data fitting in Fig. 1, Eq. (21) can be inverted to compute the overpotential $\eta$ as a function of the Helmholtz potential ${V}_{H}$, accounting for both cathodic and anodic currents. By setting $X={e}^{-\frac{F\eta }{{RT}}}$, Eq. (21) reduces to solving a second-degree polynomial.

22 $$0=-{r}_{{{\rm{H}}}+}{{{\rm{X}}}}^{2}-\frac{j}{{j}_{0}}X+{r}_{{{{\rm{H}}}}_{2}},$$

The polynomial admits a physically meaningful solution:

23 $$X={e}^{-\frac{F\eta }{{RT}}}=\frac{1}{2{r}_{{{\rm{H}}}+}}\left(\sqrt{{\left(\,\frac{j}{{j}_{0}}\right)}^{2}+4{r}_{{{\rm{H}}}+}{r}_{{{{\rm{H}}}}_{2}}}-\frac{j}{{j}_{0}}\right),$$

**Fig. 6:**
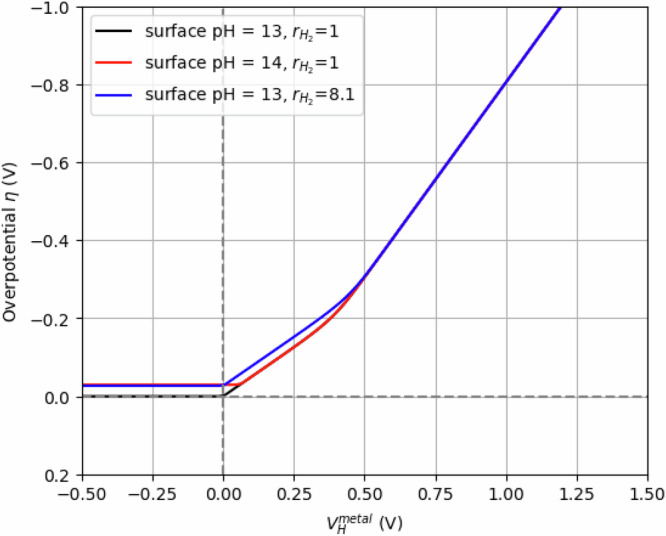
Influence of interfacial pH and H_2_ concentration on the HER overpotential. Variations in the Hydrogen Evolution Reaction (HER) overpotential ($\eta$) on metal electrodes as a function of the Helmholtz potential (${V}_{H}^{{metal}}$), depending on the surface pH and the surface-to-bulk H_2_ concentration ratio $({r}_{{H}_{2}})$. At large Helmholtz potentials, the overpotential exhibits a linear dependence on ${V}_{H}^{{metal}}$ as formally derived in the discussion. Conversely, at low overpotentials (typically < 0.43 V), the significant accumulation of H_2_ at the surface necessitates consideration of the reverse oxidation reaction, leading to a deviation from this linear relationship. In the regime of negligible or negative overpotentials, the surface proton (H^+^) concentration reaches a physical limit imposed by diffusion-limited transport and consumption during the reaction. In this limit, the overpotential plateaus at a minimum saturation value. Source data are provided as a Source Data file.

We still need to discuss the values of ${r}_{{{\rm{H}}}+}$ and ${r}_{{{{\rm{H}}}}_{2}}$. In the article, we introduce a relationship between ${r}_{{{\rm{H}}}+}$ and Helmholtz potential ${V}_{H}$, i.e., ${r}_{{{\rm{H}}}+}\propto \exp \left(-\frac{q{V}_{H}}{{k}_{B}T}\right)$. Nevertheless, at low Helmholtz potential ${V}_{H}$, it is necessary to introduce a cutoff for ${r}_{H+}$, reflecting the limitation imposed by diffusion. In other words, the surface concentration of ${{{\rm{H}}}}^{+}$ is not expected to exceed the bulk concentration due to its consumption during the reaction at the surface of the electrode. The local pH cannot be lower than the bulk pH of 13 used in these experiments. When decreasing the limit surface concentration of H^+^ from pH = 13 to pH = 14, the saturation overpotential for negative Helmholtz potential increases as shown by the red curve compared to the black curve in Fig. 6.

For ${r}_{{H}_{2}}$, a first approach consists of taking a value of 1, considering that the continuous production of ${H}_{2}$ leads to saturation of dissolved ${{{\rm{H}}}}_{2}$ in the solution, with ${\left[{{{\rm{H}}}}_{2}\right]}_{{surf}}={\left[{{{\rm{H}}}}_{2}\right]}_{{bulk}}={\left[{{{\rm{H}}}}_{2}\right]}_{{sat}}=7.8\times {10}^{-4}$ mol/L, considering the ${{{\rm{H}}}}_{2}$ solubility in water at 1 atm and 25 °C (Henry coefficient $7.8\times {10}^{-6}$ mol/m^3^/pa). In any case, the ${{{\rm{H}}}}_{2}$ concentration is not expected to depend on ${V}_{H}$. From Eq. (23), we can evaluate the ${V}_{H}$ value from which the oxidation reaction has to be taken into account. This corresponds to the ${V}_{H}$ value at which the two terms inside the square root in Eq. (23) become equal, i.e., ${\left(\frac{j}{{j}_{0}}\right)}^{2}=4{r}_{H+}{r}_{{H}_{2}}$. It occurs for ${V}_{H}=0.43$ V, as shown in Fig. 1 and in black in Fig. 6.

However, at equilibrium, an ${{{\rm{H}}}}_{2}$ concentration gradient can result from its accumulation close to the interface. Assuming a ${{{\rm{H}}}}_{2}$ current density ${j}_{{{{\rm{H}}}}_{2}}=\frac{1}{2}\left|j\right|$ at the electrode surface, resulting from its production, one can derive the concentration gradient $\left[{{{\rm{H}}}}_{2}\right]\left(z\right)$, with $z$ being the distance from the electrode into the electrolyte. We assume a planar interface, which is a reasonable approximation given the characteristic distance involved. The stationary solution $0=\frac{\partial \left[{{{\rm{H}}}}_{2}\right]}{\partial t}\left(z,t\right)$ implies that ${j}_{{{{\rm{H}}}}_{2}}\left(z\right)={j}_{{{{\rm{H}}}}_{2}}\left(0\right)=\frac{1}{2}\left|j\right|$ close to the surface, since $\frac{\partial \left[{{{\rm{H}}}}_{2}\right]}{\partial t}\left(z,t\right)=-\frac{\partial {j}_{{{{\rm{H}}}}_{2}}}{\partial z}\left(z,t\right)$. Solving the Fick law, ${j}_{{{{\rm{H}}}}_{2}}(z)=-D\frac{\partial \left[{{{\rm{H}}}}_{2}\right]}{\partial z}\left(z\right)$, with $D=4.5\times {10}^{-9}$ m^2^/s, the diffusion coefficient of ${{{\rm{H}}}}_{2}$ in water, it follows that $\left[{{{\rm{H}}}}_{2}\right]\left(z\right)=-\frac{\left|j\right|}{2D}z+{cst}.$ At a distance L, corresponding to the slipping plane, $\left[{{{\rm{H}}}}_{2}\right]\left(z\right)$ reaches a constant value due to the stirring of the solution. One can assume $\left[{{{\rm{H}}}}_{2}\right]\left(L\right)={\left[{{{\rm{H}}}}_{2}\right]}_{{sat}}$, with $L$ taken as 1 nm at molar ionic strength. It follows that $\left[{{{\rm{H}}}}_{2}\right]\left(z\right)=\frac{\left|j\right|}{2D}\left(L-z\right)+{\left[{{{\rm{H}}}}_{2}\right]}_{{sat}}$, and then

24 $${r}_{{{{\rm{H}}}}_{2}}=\frac{{\left[{{{\rm{H}}}}_{2}\right]}_{{surf}}}{{\left[{{{\rm{H}}}}_{2}\right]}_{{bulk}}}=\frac{\left[{{{\rm{H}}}}_{2}\right]\left(0\right)}{{\left[{{{\rm{H}}}}_{2}\right]}_{{sat}}}=\frac{\left|j\right|L}{2D{\left[{{{\rm{H}}}}_{2}\right]}_{{sat}}}+1,$$

This leads to ${r}_{{{{\rm{H}}}}_{2}}=8.1$. The increase in ${{{\rm{H}}}}_{2}$ concentration near the electrode leads to a slight rise in the overpotential compared to the case ${r}_{{{{\rm{H}}}}_{2}}=1$, as shown in Fig. 6 (blue curve versus black curve). For an overpotential small enough so that the H_2_ oxidation reaction becomes significant, the higher surface concentration of H_2_ enhances the reaction rate, which in turn must be balanced by a larger overpotential.

### Continuity of the electric displacement at the surface of a semiconductor electrode

When a semiconductor electrode is put in contact with a metal, having a difference of potential $\Delta {\phi }_{{SC}}$, charge transfers form a SCR with a thickness ${W}_{{SC}}$ that can be calculated simply as

25 $${W}_{{SC}}=\sqrt{\frac{2{\epsilon }_{0}{\epsilon }_{r}^{{SC}}}{q{N}_{D}}\Delta {\phi }_{{SC}}}$$

with ${\epsilon }_{r}^{{SC}}$ and ${\epsilon }_{0}$ the relative permittivity of the semiconductor and of the vacuum, respectively, and ${N}_{D}$ the density of charge carriers.

Considering the continuity of the electric displacement at the interface, the Helmholtz potential can be derived as:

26 $${V}_{H}=\frac{{\epsilon }_{r}^{{SC}}}{{\epsilon }_{r}^{{HL}}}\frac{{W}_{{HL}}}{{W}_{{SC}}}\Delta {\phi }_{{SC}},$$

with ${\epsilon }_{r}^{{SC}}$ and ${\epsilon }_{r}^{{HL}}$ the relative permittivity of the semiconductor and the Helmholtz layer, respectively, and ${W}_{{HL}}$ the width of the Helmholtz layer. The relative permittivity of the electrolyte remains close to that of water (${\epsilon }_{r}^{{HL}}\sim 80$) even if it decreases with increasing ion concentration^25^, so that ${\epsilon }_{r}^{{SC}} < {\epsilon }_{r}^{{HL}}$ and ${W}_{{SC}} > > {W}_{{HL}}$ for a classical semiconductor in concentrated electrolyte (>0.1 M). Interestingly, ${V}_{H}$ is therefore negligible for semiconductors.

In Eq. (26), surface charges are not explicitly considered, which may seem surprising as surface states and defects often trap charges and ions can absorb. This omission is justified by the fact that the potential drop is attributed to the charge distribution within the Helmholtz layer, which inherently includes any surface charges. Alternatively, since the Helmholtz layer is extremely thin at high ionic strength, its total charge can be effectively treated as a surface charge. The results presented here remain unchanged whether one interprets the effect as the energy required for a reactant to adsorb or desorb from a charged surface, or as the energy needed to overcome the potential barrier across the Helmholtz layer.

### Derivation of the evolution of the potential in a semiconductor thin film

The evolution of the potential $\phi (x)$ in the semiconductor is given by the Poisson equation with a uniform charge density $q{N}_{D}$ as described as:

27 $$\frac{{d}^{2}\phi }{d{x}^{2}}=-\frac{q{N}_{D}}{{\epsilon }_{0}{\epsilon }_{r}^{{SC}}}$$

Solving this equation considering $\phi (x=0)={WF}/q$ and $\phi (x=d)=-\mu /q-{V}_{H}$, leads to:

28 $$\phi \left(x\right)=-\frac{q{N}_{D}}{2{\epsilon }_{0}{\epsilon }_{r}^{{SC}}}{x}^{2}+{C}_{1}x+{WF}/q$$

with:

29 $${C}_{1}=\frac{q{N}_{D}}{2{\epsilon }_{0}{\epsilon }_{r}^{{SC}}}d-\frac{\mu+{WF}+q{V}_{H}}{{qd}}$$

At the interface between the electrolyte and the semiconductor, the continuity of the electric displacement gives:

30 $${\epsilon }_{0}{\epsilon }_{r}^{{SC}}\left(-\frac{q{N}_{D}}{{\epsilon }_{0}{\epsilon }_{r}^{{SC}}}d+{C}_{1}\right)={\epsilon }_{0}{\epsilon }_{r}^{{HL}}\frac{{V}_{H}}{{W}_{{HL}}}.$$

Combining both equation leads to an expression of the Helmholtz potential:

31 $$q{V}_{H}=\frac{1-{\left(\frac{d}{{W}_{{SC}}}\right)}^{2}}{1+\frac{{\epsilon }_{r}^{{HL}}}{{\epsilon }_{r}^{{SC}}}\frac{d}{{W}_{{HL}}}}\left[-\mu -{WF}\right],$$

where

32 $${W}_{{SC}}=\sqrt{\frac{2{\epsilon }_{0}{\epsilon }_{r}^{{SC}}}{{q}^{2}{N}_{D}}\left[-\mu -{WF}\right]}$$

would be the width of the SCR for the potential difference $-(\mu+{WF})/q$ defined when ${V}_{H}=0$. Note that $\left[-\mu -{WF}\right]$ is only positive in the limit of the model ${WF} < -\mu$.

Compared to Eq. (3), a dependence of the Helmholtz potential linear with the difference $-\mu -{WF}$, similar to the one derived for metal, is observed, except that the coefficient $\kappa$ tends to zero as *d* approaches the value ${W}_{{SC}}$.

### Consideration on the resistivity of the semiconducting thin film

The presence of the thin hydroxyl layer can introduce an additional resistive component to the system, effectively behaving as a series resistor and contributing to the observed increase of $\eta$ by:

33 $${\eta }_{R}=\frac{j}{q{\mu }_{n}{N}_{D}}d.$$

with $j$ the current density and ${\mu }_{n}$ the charge mobility. As discussed previously, increasing the density of charge carriers and decreasing the thickness can help to mitigate this potential drop. But this overpotential remains negligible even for a low amount of charge carriers and large current densities relevant for industrial applications, and a thickness of hundreds of nanometers.

## Supplementary information

Supplementary Information

Transparent Peer Review file

## Source data

Source Data

## Data Availability

All experimental data presented in this study are sourced from the literature and summarized in the Supplementary Information tables. Source data are provided with this paper.
