# Supplementary material for: The role of the Helmholtz potential on electrocatalytic activity
Source: Nat Commun. 2026 Mar 28;17:4547. doi: 10.1038/s41467-026-70980-5 (PMC13195119; doi:10.1038/s41467-026-70980-5)
Supplement: Supplementary file 1 — Supplementary Information [file 41467_2026_70980_MOESM1_ESM.pdf]

Supplementary Information

The role of the Helmholtz potential on electrocatalytic activity

Arsène Chemin[1*], Louis Godeffroy[2], David Amans[1], Tristan Petit[2]

1 Univ. Lyon, Université Claude Bernard Lyon 1, CNRS, UMR5306, Institut Lumière Matière, Villeurbanne, F-69100, France

2 Helmholtz-Zentrum Berlin für Materialien und Energy GmbH Nanoscale Solid-Liquid Interfaces Albert-Einstein Str. 15 Berlin Germany

* Corresponding author: arsene.chemin@univ-lyon1.fr

Supplementary Note 1: Effective carrier density and experimental feasibility of ultrathin semiconductor layers

In this work, the carrier concentration range discussed for ultrathin hydroxide semiconductor layers corresponds to an effective carrier density rather than to conventional substitutional bulk doping. This distinction is particularly important for materials such as $Ni(OH)_2$, $Co(OH)_2$, and FeOOH, whose electronic properties are predominantly governed by native defects (e.g., oxygen vacancies, interstitial species) and localized charge carriers, rather than by well-defined substitutional dopants. The lower bound of the proposed carrier density range is consistent with experimentally reported intrinsic carrier concentrations in these materials, while higher effective densities can be achieved through defect engineering and transition-metal incorporation. In addition to intrinsic defects, the electronic properties of $Ni(OH)_2$, $Co(OH)_2$, and FeOOH can be tuned through metal incorporation, such as Fe in $Ni(OH)_2$, Ni in $Co(OH)_2$, or Ni in FeOOH. These approaches have been shown to significantly modify carrier densities, conductivity, and electrochemical activity, without requiring ideal crystalline order.

Electrochemical synthesis routes are particularly well suited for implementing this strategy and were employed in the experimental results discussed in the main text. It enables the growth of ultrathin films and nanostructured islands with controlled morphology and composition, without the need for atomically precise substitutional doping. Importantly, effective carrier densities can be tuned by adjusting electrochemical parameters such as applied potential, current density, electrolyte composition, and pH, which directly influence defect formation and charge compensation mechanisms. For instance, Fe-doped $Ni(OH)_2$ films prepared by electrochemical deposition have been reported to exhibit large effective carrier densities, with Fe concentrations reaching up to ~16 at.%.[1] Moreover, the simplicity, scalability, and industrial compatibility of electrochemical methods make them particularly attractive for practical applications.

Alternative thin-film fabrication techniques can also provide access to comparable carrier density ranges while offering different levels of control. Pulsed Laser Deposition (PLD) and Chemical Vapor Deposition (CVD) allow for high stoichiometric precision and controlled incorporation of dopants but require complex infrastructure and are less readily scalable. Atomic Layer Deposition (ALD) and sputtering enable uniform films and controlled compositions, albeit at the expense of slower deposition rates or higher equipment costs. Chemical Bath Deposition (CBD) provides a low-cost route to hydroxide films with limited control over doping, while sol–gel methods offer a versatile and widely used wet-chemical approach for producing homogeneous oxide and hydroxide films with tunable composition and morphology. Across these techniques, effective carrier densities on the order of 10^{17}–10^{20} cm^{-3} are commonly reported, with material-specific variations.

Supplementary Table 1: HER overpotential for metal electrodes

HER overpotential values, measured at -5 mA cm^{-2} in 0.1 M $HClO_4$ and 0.1 M KOH, were taken from Danilovic et al[2] and Dadallagei et al[3]. WF values were taken from Sheng et al[4]. The polycrystalline materials were considered.

**Metal**	**WF (eV)**	**-η_{HClO_4} (V)**	**-η_{KOH} (V)**	**-η_{KOH} (V)**
Cu	4.51	0.42	0.53	0.60
Ag	4.39	0.40	0.60	0.64
Au	5.30	0.33	0.62	0.63

Ru	4.71	0.18	0.24	/
Ir	5.28	0.02	0.04	/
Pt	5.30	0.02	0.11	0.07
V	4.10	0.73	0.78	/
Ti	3.87	0.87	0.98	/
Ni	5.06	0.27	0.31	0.33
W	4.56	/	/	0.44
Co	4.71	/	/	0.36
Fe	4.55	/	/	0.35
Pd	5.17	/	/	0.31

Supplementary Table 2: HER overpotential for metal electrodes covered by a thin film of nickel hydroxide

HER overpotential values, measured at -5 mA cm^{-2} in 0.1 M KOH, were taken from Danilovic et al[2]. WF values were taken from Sheng et al[4]. The polycrystalline materials were considered.

**Metal**	**WF (eV)**	**-η_{bare} (V)**	**-$\eta_{Ni(OH)2}$ (V)**
Cu	4.51	0.53	0.44
Ag	4.39	0.60	0.42
Au	5.30	0.62	0.38
Ru	4.71	0.24	0.22
Ir	5.28	0.04	0.02
Pt	5.30	0.11	0.04

V	4.10	0.78	0.56
Ti	3.87	0.98	0.60
Ni	5.06	0.31	0.24

Supplementary Table 3: HER overpotential for Pt(111) electrodes covered by a thin film of hydroxide

HER overpotential values, measured at -5 mA cm^{-2} in 0.1 M KOH, were taken from Subbaraman et al.[5]. The WF value of Pt(111), 5.84 eV, was taken from Sheng et al.[4]. The electronic properties of 2D $Ni(OH)_2$, 2D $Co(OH)_2$ and nanosized FeOOH were taken from refs.[6,7], ref.[8] and ref.[9], respectively. The bandgap energy of 2D $Co(OH)_2$ was extrapolated from the Tauc plots based on Ping et al.[7]. In the case of nanosized FeOOH, the γ/δ polymorphs were considered.

**Hydroxide**	**Bandgap (eV)**	**EA (eV)**	$\boldsymbol{E_i}$ **(eV)**	**-$\boldsymbol{\eta}$ (V)**
$Ni(OH)_2$	2.54	1.47	4.01	0.03
$Co(OH)_2$	2.20	2.93	5.13	0.12
FeOOH	/	/	6.00	0.27

References

1. Wang, Z., Li, Z., Chao, Y., Cui, Y., He, X., Liang, P., ... & Zhang, Z. Dealloying-derived Fe-doped Ni (OH) 2/Ni foils as self-supported oxygen evolution reaction catalysts. *J. Energy Mater.* 2 (2022): 200019.

2. Danilovic, N. *et al.* Enhancing the alkaline hydrogen evolution reaction activity through the bifunctionality of $Ni(OH)_2$/metal catalysts. *Angewandte Chemie - International Edition* **51**, 12495–12498 (2012).

3. Dadallagei, K. S. R. *et al.* New Perspectives from Classical Transition State Theory: The Hydrogen Evolution Reaction on Metal Electrodes. *Journal of The Electrochemical Society* **170**, 086508 (2023).

4. Sheng, W., Myint, M., Chen, J. G. & Yan, Y. Correlating the hydrogen evolution reaction activity in alkaline electrolytes with the hydrogen binding energy on monometallic surfaces. *Energy & Environmental Science* **6**, 1509–1512 (2013).

5. Subbaraman, R. *et al.* Trends in activity for the water electrolyser reactions on 3d M(Ni,Co,Fe,Mn) hydr(oxy)oxide catalysts. *Nature Materials* **11**, 550–557 (2012).

6. Kim, S. I., Thiyagarajan, P. & Jang, J. H. Great improvement in pseudocapacitor properties of nickel hydroxide via simple gold deposition. *Nanoscale* **6**, 11646–11652 (2014).

7. Ping, L. *et al.* Synthesis of 2D layered transition metal (Ni, Co) hydroxides via edge-on condensation. *Scientific Reports* **14**, 1–9 (2024).

8. Suksomboon, M., Kongsawatvoragul, K., Duangdangchote, S. & Sawangphruk, M. Reducing the Energy Band Gap of Cobalt Hydroxde Nanosheets with Silver Atoms and Enhancing Their Electrical Conductivity with Silver Nanoparticles. *ACS Omega* **6**, 20804–20811 (2021).

9. Ali, F. M., Hmadeh, M., O’Brien, P. G., Perovic, D. D. & Ozin, G. A. Photocatalytic Properties of All Four Polymorphs of Nanostructured Iron Oxyhydroxides. *ChemNanoMat* **2**, 1047–1054 (2016).
