# Supplementary material for: The role of the Helmholtz potential on electrocatalytic activity
Source: Nat Commun. 2026 Mar 28;17:4547. doi: 10.1038/s41467-026-70980-5 (PMC13195119; doi:10.1038/s41467-026-70980-5)
Supplement: Supplementary file 2 — Transparent Peer Review file [file 41467_2026_70980_MOESM2_ESM.pdf]

nature portfolio

Peer Review File

The role of the Helmholtz potential on electrocatalytic activity

Corresponding Author: Dr Arsène Chemin

This file contains all reviewer reports in order by version, followed by all author rebuttals in order by version.

Version 0:

Reviewer comments:

Reviewer #1

(Remarks to the Author)
Comments to the Author:
This manuscript presents a valuable theoretical framework that integrates the Helmholtz potential into the Butler-Volmer formalism to explain electrocatalytic activity, with a focus on the hydrogen evolution reaction. The proposed model, which describes the co-regulation of kinetics by the Sabatier principle and the Helmholtz potential, and the role of semiconductor thin films in modulating the interfacial potential, is benefit for the rational design of advanced electrocatalysts. While the topic of this manuscript may be of interest to “Nature Communications” readers, a few points may need to be addressed. Please see below for details：

Q1. The central parameter κ is empirically fitted as 0.4 for Ni(OH)2. For broader utility, could the authors clarify the calculation of κ, including the values chosen for key parameters like the permittivity of the semiconductor and the Helmholtz layer? Furthermore, does a general relationship exist between κ and semiconductor thickness across different materials (e.g., $Co(OH)_2$, FeOOH)? A brief quantitative discussion would greatly enhance clarity.

Q2. The proposed optimal film thickness of 1-10 nm appears to be a general recommendation. How does the work function of the underlying metal substrate influence this optimal range? For instance, would a low-WF metal (e.g., Ti) require a different optimal thickness compared to a high-WF metal (e.g., Pt) to achieve the same κ? A discussion on this interplay would provide more precise design guidelines.

Q3. The model's fit to acidic HER data relies on an assumed local pH of ~10. Could the authors comment on how the presence of a semiconductor overlayer (e.g., Ni(OH)2, which can interact with OH-) might further perturb the local pH? Clarifying whether this effect is implicitly accounted for or should be considered a secondary factor would improve the model.

Q4. The proposed physical limit of 10 A·cm-2 for the HER exchange current density is a key finding. Could the authors please specify the boundary conditions of this limit? For example, is it expected to hold for non-traditional catalysts or under non-ambient conditions?

Q5. Could you explicitly elaborate on the physical origin of the dual criterion ((Ei < WF) and (Ei < -μ)) for optimizing interfacial charge transfer and Helmholtz potential modulation? To enhance readers’ intuitive understanding, a schematic diagram should be provided. For instance, what specific interfacial charge behaviors (e.g., carrier accumulation/depletion in the semiconductor’s space-charge region, or alignment of energy levels between the metal, semiconductor, and electrolyte) would be disrupted if only one condition is satisfied?

Q6. The ionization energy is highlighted as a critical property, but a systematic comparison is lacking. Providing a supplementary table that lists Ei, experimentally derived κ, and the resultant change in overpotential for known semiconductors (Ni(OH)2, Co(OH)2, FeOOH) would powerfully illustrate the correlation and strengthen the rationale for material selection.

Q7. In the “conditions of interest” region of Fig. 2c, the doping concentration of the semiconductor thin film is specified to 1017-21 cm−3 under the simultaneous constraint of a thin film thickness (1-10 nm). To enhance the practical relevance and credibility of your proposed semiconductor design strategy, it would be valuable for the authors to provide illustrative examples and experimental substantiation addressing the achievable doping concentrations of Ni(OH)2, Co(OH)2, FeOOH

noted in the study.

Reviewer #2

(Remarks to the Author)
The manuscript proposes an electrostatic viewpoint for electrocatalytic kinetics by explicitly accounting for the Helmholtz potential drop and its impact on the near surface concentration of reactants and intermediates. The authors argue that this effect can be used to interpret polarization behavior and to derive design guidelines, including the use of ultrathin semiconductor layers to reduce the Helmholtz potential penalty. The topic is interesting and the framework could be useful for thinking about kinetics beyond standard Butler–Volmer treatments. I have a few minor points that would improve clarity and help readers judge how broadly the approach can be applied.

1. Your framework is formulated in terms of work function and Helmholtz potential. How do you expect it to carry over to practical catalyst surfaces such as alloys, high entropy alloys, oxides, and carbon supported catalysts, where reconstruction and partial oxidation under bias are common?

2. Beyond HER, how would you write the same treatment for reactions such as ORR, HOR, or CO2RR? Which steps or intermediates do you expect to be most sensitive to the Helmholtz potential, and how would you implement the surface concentration correction for charged or strongly polar intermediates?

3. In cases where mass transport limitations are significant, how do you separate diffusion driven concentration polarization from the Helmholtz potential driven surface concentration effect when fitting polarization curves?

4. How robust are the thin double layer and linear Helmholtz drop assumptions in non aqueous electrolytes, ionic liquids, or water in salt systems, where dielectric properties and interfacial structure can differ substantially? What modifications would be needed for those cases?

5. For the ultrathin semiconductor layer concept, can you provide more concrete selection criteria and boundary conditions, for example thickness window, band alignment requirements, and the tradeoff between reducing the Helmholtz potential and maintaining access to metallic active sites for different reactions?

Reviewer #3

(Remarks to the Author)
In this work, Chemin et al. propose a hypothesis linking the Helmholtz potential to the electrocatalytic activity of the hydrogen evolution reaction (HER). Specifically, the authors define the Helmholtz potential as the potential drop at the electrode-electrolyte interface, which arises from the energy difference between the work function of the electrode and the chemical potential of the electrolyte. They argue that this potential drop induces a rigid water structure at the electrode interface and reduces the local proton concentration due to the associated electric field, thereby affecting HER kinetics. To support their hypothesis, the authors performed numerical simulations based on the Butler-Volmer equation, incorporating the effect of the Helmholtz potential. These simulations were compared with experimental data previously reported in the literature, and the model showed good agreement, which the authors present as validation of their hypothesis. Furthermore, they explain the enhanced HER performance in alkaline media observed upon the addition of metal hydroxides to the catalyst surface as a result of a reduced Helmholtz potential. This study introduces a novel conceptual framework to the HER research community. However, the reviewer finds that some of the key assumptions underlying the hypothesis are not adequately justified and appear to overlook fundamental principles established in HER studies. Therefore, the validity of the proposed hypothesis remains uncertain, which this reviewer believes that the manuscript may not meet the standards required for publication in Nature Communications.
Comments
1) The authors describe HER activity using a Butler-Volmer equation based on the proton reduction reaction, which is appropriate under acidic conditions where protons are readily available at the electrode surface. However, they extend this model to alkaline conditions, where the dominant HER mechanism involves water reduction. This mechanistic discrepancy may be significant, as the kinetics and interfacial phenomena governing HER in alkaline media differ fundamentally from those in acidic environments. Consequently, the direct application of their model to alkaline HER is not justified, casting doubt on the validity of the proposed hypothesis in such conditions.
2) In their numerical simulation of HER under acidic conditions, the authors fixed the local pH at 10 to illustrate the effect of the Helmholtz potential. However, if the local pH were indeed 10, the HER mechanism would be expected to shift from proton reduction to water reduction, leading to pH-independent kinetics. Such behavior, however, is not observed in the experimental data in acid electrolytes (Nat. Chem. 2013, 5, 300), suggesting that the assumption of a fixed local pH at this value may be flawed. Furthermore, the assumption of a constant local pH contradicts the authors' own argument that the reduced HER activity arises from a decrease in local proton concentration, which is strongly influenced by the Helmholtz potential. Therefore, the local pH should not be treated as constant but rather as a dynamic variable dependent on the Helmholtz potential. This relationship should be explicitly incorporated into the Butler-Volmer equation used in their model.
3) By estimating the proton consumption rate at a current density of -5 mA/cm2, the corresponding rate (j/F) is calculated to be 5.2 * 10^(-8) mol/s/cm2. In comparison, the maximum proton diffusion flux in 0.1 M HClO4—using a proton diffusion coefficient of 9.3 * 10^(-5) cm2/s, a diffusion layer thickness of 0.96 nm (estimated from the Debye length), and calculation by the Fick's 1st law—is approximately 0.097 mol/s/cm2. This diffusion flux is about 1.8 * 10^(6) times greater than the calculated proton consumption rate. Such a large discrepancy indicates that the proton supply far exceeds the consumption,

making it numerically implausible to justify the elevated local pH value assumed by the authors. This inconsistency further undermines the validity of their model.

Version 1:

Reviewer comments:

Reviewer #1

(Remarks to the Author)
The author replied to my question and agreed to publish.

Reviewer #2

(Remarks to the Author)
The author has already answered my question, and I recommend publishing it.

Reviewer #3

(Remarks to the Author)
All questions raised by the reviewer have been greatly addressed. No more questions or comments from this reviewer.

Open Access This Peer Review File is licensed under a Creative Commons Attribution 4.0 International License, which permits use, sharing, adaptation, distribution and reproduction in any medium or format, as long as you give appropriate credit to the original author(s) and the source, provide a link to the Creative Commons license, and indicate if changes were made.
In cases where reviewers are anonymous, credit should be given to 'Anonymous Referee' and the source.
The images or other third party material in this Peer Review File are included in the article’s Creative Commons license, unless indicated otherwise in a credit line to the material. If material is not included in the article’s Creative Commons license and your intended use is not permitted by statutory regulation or exceeds the permitted use, you will need to obtain permission directly from the copyright holder.
To view a copy of this license, visit https://creativecommons.org/licenses/by/4.0/

Dear Reviewers,

We thank you and the three reviewers for the careful evaluation of our manuscript and for the constructive and insightful comments. We are grateful for the opportunity to clarify the scope of our theoretical framework, to better justify the underlying assumptions, and to strengthen the manuscript through additional discussion, and supplementary material. Below we provide a point-by-point response to each reviewer. All changes made to the manuscript are clearly indicated in the revised version in yellow.

First of all, we would like to point out, we found that there was a mistake in the numbering of the equations, with two equation (8) and we apologies for it. As the consequence, the number of the equations (8) bis to (10) have been shifted to (9) to (11).

We also spotted a typo in the Figure 2b and we replace $\frac{\mu - WF}{q}$ by $-\frac{\mu + WF}{q}$ as it is defined in the text.

Response to Reviewer #1

We thank Reviewer #1 for the positive assessment of the conceptual framework and for the detailed questions, which help clarify the physical interpretation and practical applicability of the model.

Q1. Clarification of the parameter κ and its calculation

Question: The central parameter κ is empirically fitted as 0.4 for Ni(OH)2. For broader utility, could the authors clarify the calculation of κ, including the values chosen for key parameters like the permittivity of the semiconductor and the Helmholtz layer? Furthermore, does a general relationship exist between κ and semiconductor thickness across different materials (e.g., $Co(OH)_2$, FeOOH)? A brief quantitative discussion would greatly enhance clarity.

Response: We agree that the experimental calculation of the parameter κ from the experimental data presented in figure 3d should be clarified.

The calculation of κ is defined in the equation (9) and its theoretical expression is given in the equation (10) from the main text. The full derivation of the expression from the Poisson equation is provided in the Supplementary Note 3 as indicated in the main text. In the case of the $Ni(OH)_2$ thin layer, we indeed fitted its value on the experimental data presented in figure 3d and it corresponds simply to the slope of the linear regression. Indeed, as we previously established in the eq. 8, the overpotential $-\eta$ is proportional to the Helmholtz potential in the system which is in our case V_H^{SC}:

$$-\eta = V_H^{SC} - \eta_0$$

Which can be rewritten as a function of κ using eq. 9:

$$-\eta = \kappa V_H^{metal} - \eta_0$$

To clarify these points, we added in the section 3.3 page 15:

"A value of $\kappa = 0.4$ can be extracted from these experimental data by considering the slope of the corresponding linear regression. As shown previously (eq. 8), the overpotential $-\eta$ scales linearly with the Helmholtz potential of the system, which in the present case corresponds to V_H^{SC}. Using the definition of κ introduced in eq. 9, this relationship can be rewritten as $-\eta = \kappa V_H^{metal} - \eta_0$."

The general relationship that exists between κ and the semiconductor thickness across different materials is given by the eq. 10. and is discussed in the section 3.2 and figure 2 for typical values of $\epsilon_r^{HL} \simeq 80$ and $\epsilon_r^{SC} \simeq 20$ and depending on the dopant concentration, thickness, and Helmholtz layer thickness. Accounting for the reviewer comment, we added in this section a brief quantitative discussion for the estimation of these parameters p. 12:

"In practice, W_{HL} can be estimated by the Debye length in the electrolyte depending on the ion concertation (typically 1 nm at 0.1 M). The relative permittivity of the electrolyte ϵ_r^{HL} usually remains close to that of bulk water so that $\epsilon_r^{HL} \simeq 80$, although it is known to decrease slightly with increasing ionic strength.[32] The relative permittivity of the thin layer ϵ_r^{SC} is material-dependent."

As for the parameters used in the theoretical estimation of $\mathrm{d} \sim 0.5$ nm derived from the eq. 10, this value was intended as an order-of-magnitude estimate, given the substantial uncertainty in the relative permittivity of the hydroxide layer. In practice, the permittivity of such layers can vary from values as low as ∼4 up to several tens,[33] depending on factors such as oxidation state, structural disorder, and water uptake. For the purpose of this estimate, we adopted an intermediate value of $\varepsilon_r^{SC} \simeq 25$. In light of the reviewer's comment, we have taken this opportunity to extend the discussion and strengthen the comparison between theory and experiment by directly extracting the effective permittivity from the experimental data as well as positioning a "star" in the **figure 2c** that corresponds to the expected conditions obtained by Markovic and co-workers. This analysis is now presented in **Section 3.3 (page 15)**:

"The value of κ can be derived theoretically from eq. 10 considering $d \ll W_{SC}$. The thickness of the Helmholtz layer is estimated to be on the order of 1 nm, consistent with the Debye length expected for electrolytes at a concentration of 0.1 M under the experimental conditions considered here. At these concentrations, the relative permittivity of the electrolyte remains close to that of bulk water ($\epsilon_r^{HL} \simeq 80$).[32] Determining the permittivity of the $Ni(OH)_2$ thin film is more complex. Pure nickel hydroxide electrode can have a permittivity as low as 4 that increases up to several tens because of oxidation and water absorption, for example after cycling in an alkaline cell [33]. In practice, the porous nature of the clusters and their partial infiltration by the electrolyte further increases the relative permittivity of the thin layer. Assuming a thickness $d = 0.7$ nm for the $Ni(OH)_2$ clusters on Pt(111), as reported from STM measurements,[2] and the value $\kappa \simeq 0.4$, the corresponding permittivity of the $Ni(OH)_2$ thin film is estimated to be $\epsilon_r^{SC} \simeq 37$. Furthermore, for sub-nanometric layers, other factors such as electron transfer through tunnelling should also be considered, which would lead to a behavior closer to that of the bare metal electrode ($\kappa = 1$), thereby increasing the experimentally observed value of κ and the estimated value of ϵ_r^{SC}."

With the reference:

33. Motori, A., Sandrolini, F., & Davolio, G. Electrical properties of nickel hydroxide for alkaline cell systems. Journal of power sources, 48(3), 361-370. (1994).

Q2. Influence of the metal substrate work function on optimal thickness

Question: The proposed optimal film thickness of 1-10 nm appears to be a general recommendation. How does the work function of the underlying metal substrate influence this optimal range? For instance, would a low-WF metal (e.g., Ti) require a different optimal thickness compared to a high-WF metal (e.g., Pt) to achieve the same κ? A discussion on this interplay would provide more precise design guidelines.

Response: According to the model eq. 10, the optimal thickness, leading to $\kappa = 0$ and fully cancelling the Helmholtz potential, appears to be W_{SC} as we can observe in the Figure 2c along the red line. Indeed,

it depends on V_H^{metal} through the expression of W_{SC} and thus on the difference between the work function of the underlying metal and the chemical potential of the electrolyte. A low-WF metal (e.g., Ti) would imply a thicker optimal thickness to counter the large amount of charge accumulated at the surface of the metal.

However, in practice, is it superfluous to optimize this thickness to match W_{SC}. In the first place it can be difficult to calculate W_{SC} which depends on ϵ_r^{SC} and N_D which can be hard to determine and control. Also, increasing too much the thickness of the layer to match W_{SC} can lead to other limitation in the charge transport as we discuss in the article. But mostly, it is easy to archive $\kappa \sim 0$ with thin layer having $d \ll W_{SC}$ as we can see in the Figure 2c. In that case the eq. 10 simplify so that κ does not depends on W_{SC} anymore. Strong reduction of the Helmholtz potential larger than 90% can be achieved with the simple general recommendation we make, regardless of more complicated recommendations.

The work function of the metal substrate does, however, determine the absolute magnitude of the remaining Helmholtz potential for a given κ (see eq. 9). A better initial match between the metal work function and the electrolyte chemical potential is always recommended to obtain smaller Helmholtz potential.

We modified the discussion of this at the end on the section 3.2 p 13.

"While the theoretically optimal film thickness to fully cancel the Helmholtz potential is $d = W_{SC}$, achieving this condition in practice is challenging, as W_{SC} depends on both the semiconductor dielectric constant ϵ_r^{SC} and the charge carriers' density N_D, which are often difficult to determine accurately and to control experimentally. Moreover, increasing the film thickness to match W_{SC} in the low-doping regime can introduce additional limitations to charge transport. In the limit $N_D \to 0$, the activity of the electrode is limited by the resistivity of the film and the low interface charge carrier density enabling the reaction (see Supplementary Note 4). Importantly, a strong reduction of the Helmholtz potential exceeding 90% can already be achieved with thin layers satisfying $d \ll W_{SC}$, as shown in Fig. 2c."

Q3. Local pH perturbation by semiconductor overlayers

Question: The model's fit to acidic HER data relies on an assumed local pH of ~10. Could the authors comment on how the presence of a semiconductor overlayer (e.g., Ni(OH)2, which can interact with OH-) might further perturb the local pH? Clarifying whether this effect is implicitly accounted for or should be considered a secondary factor would improve the model.

Response: We agree that semiconductor overlayers such as $Ni(OH)_2$ can locally interact with OH^- species. At the very beginning of the experiment, when only a small number of protons have been consumed and the local pH remains close to the bulk value (≈1), the $Ni(OH)_2$ layer can indeed have a slight influence on the local pH, as it partially dissolves and releases OH^- ions. However, as the hydrogen evolution reaction (HER) proceeds, the local pH rapidly increases above ≈8–9, a range in which $Ni(OH)_2$ is thermodynamically stable and therefore no longer affects the local pH. Consequently, $Ni(OH)_2$ does not significantly influence the local pH evolution, since the HER itself induces a much larger and dominant increase in pH. It is nevertheless worth noting that it is precisely this HER-induced local basification of the electrolyte that allows such metal/metal hydroxide heterostructures to remain stable, even under strongly acidic bulk conditions.

Regarding the model itself, the pH we consider to determine the chemical potential of the electrolyte and the Helmholtz layer is the one away from the surface, at a few Debye layers (typically a few nm) and would not be influenced by the chemical interaction of the OH^- species with the $Ni(OH)_2$ surface but only by the local production and diffusion of OH^- species as discussed p9.

For further discussion on the evolution of the pH, one can refer to Questions 1 and 3 raised by Referee 3, as well as to the corresponding discussion added to the Supplementary Materials 0 and the modifications made to the main text.

Q4. Boundary conditions of the proposed exchange current density limit

Question: The proposed physical limit of 10 A·cm-2 for the HER exchange current density is a key finding. Could the authors please specify the boundary conditions of this limit? For example, is it expected to hold for non-traditional catalysts or under non-ambient conditions?

Response: The physical limit of 10 A·cm-2 for the HER exchange current corresponds by definition to the value of j_0 determined from the eq. 7 considering the measured η_0 from the Figure 1. One can also notice a small dispersion of the measured overpotential compared to the model in the Figure 1 due either to experimental variability or catalytic properties of the various metal surfaces which is not encompassed by this model. As such it corresponds to a limit for these experimental conditions, i.e. the average behavior of metal electrodes under ambient conditions. For non-traditional catalysts or under non-ambient conditions, one can hope to push further this limit.

We clarify this by adding a brief discussion p.9:

"It can be interpreted as the physical limit for the exchange current density for the HER, under the considered experimental conditions and for the metals studied, assuming ideal diffusion of protons to the electrode surface and no reverse reactions. Pushing beyond this limit would require considering other types of catalysts or non-ambient conditions."

Q5. Physical origin of the dual criterion involving ionization energy

Question: Could you explicitly elaborate on the physical origin of the dual criterion ((Ei < WF) and (Ei < -µ)) for optimizing interfacial charge transfer and Helmholtz potential modulation? To enhance readers' intuitive understanding, a schematic diagram should be provided. For instance, what specific interfacial charge behaviors (e.g., carrier accumulation/depletion in the semiconductor's space-charge region, or alignment of energy levels between the metal, semiconductor, and electrolyte) would be disrupted if only one condition is satisfied?

Response: The discussion of the dual criterion (Ei < WF and Ei < −µ) is indeed particularly insightful, as it provides a rational explanation for the differing performances observed for $Ni(OH)_2$, $Co(OH)_2$, and FeOOH. In the original manuscript, this discussion was presented in Supplementary Note 5, including the requested schematic diagram; however, in light of the reviewer's interest, we have moved it to the main text.

The physical origin of this criterion comes from the fact that when both conditions Ei < WF and Ei < −µ are satisfied, the Fermi level lies below the valence band maximum of the semiconductor layer, leading to a deep depleted space-charge region. As a consequence, the electrical conductivity of the layer is significantly enhanced, thereby facilitating charge transfer across the interface. This situation applies to $Ni(OH)_2$ and, to a lesser extent, to $Co(OH)_2$, whereas it is not fulfilled for FeOOH. Consequently, Pt(111)/$Ni(OH)_2$ is more active than Pt(111)/$Co(OH)_2$, and Pt(111)/FeOOH is even less active than bare Pt(111), FeOOH blocking some active sites. To clarify this mechanism, a new schematic figure (Figure 4) has been added, illustrating the energy-level alignment, space-charge formation, and interfacial charge accumulation, and directly relating these features to the measured overpotentials.

New figure 4:

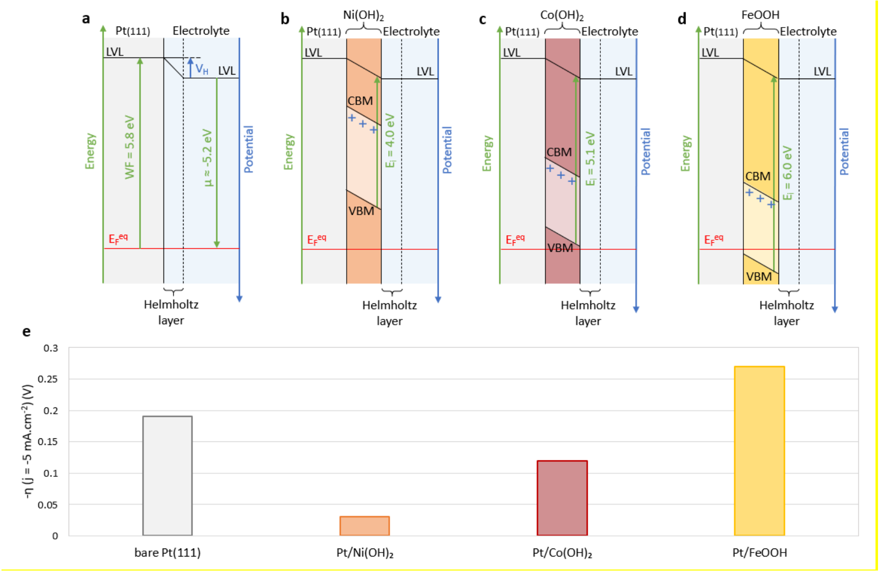

Figure 4: Modulation of the HER overpotential on Pt(111) surface by a thin metal hydroxide layer. Band alignment and interfacial equilibrium for a Pt(111) surface (a) bare, covered with a thin film of (b) $Ni(OH)_2$, (c) $Co(OH)_2$, and (d) FeOOH. Trapped charges from *n*-type dopants are indicated by blue "+" signs. (e) HER overpotential values of these surfaces measured at -5 mA cm^{-2} in 0.1 M KOH, taken from ref. [5]. The WF value of Pt(111) was taken from ref. [20]. The electronic properties of 2D $Ni(OH)_2$, 2D $Co(OH)_2$ and nanosized FeOOH were taken from refs. [26,27], ref. [28] and ref. [29], respectively. The bandgap energy of 2D $Co(OH)_2$ was extrapolated from the Tauc plots based on ref. [27]. In the case of nanosized FeOOH, the γ/δ polymorphs were considered. All values are summarized in Supplementary Table 3.

Q6. Systematic comparison of ionization energy and κ

Question: The ionization energy is highlighted as a critical property, but a systematic comparison is lacking. Providing a supplementary table that lists Ei, experimentally derived κ, and the resultant change in overpotential for known semiconductors (Ni(OH)2, Co(OH)2, FeOOH) would powerfully illustrate the correlation and strengthen the rationale for material selection.

Response: We agree that a more systematic comparison strengthens the manuscript. We have therefore added a comparison of the ionization energies of $Ni(OH)_2$, $Co(OH)_2$, and FeOOH supported on Pt(111), referenced systematically to the Pt(111) surface following the approach of Subbaraman et al.[5] These values are compared with the corresponding measured overpotentials in Supplementary Table 3 and are further discussed in relation to our model in the newly added Figure 4.

While the ionization energy plays a key role in determining whether the dual criterion is satisfied, it does not directly influence the value of κ. In our framework, κ is governed by the electrostatic screening of the Helmholtz potential through charge accumulation within the thin layer by the charge carriers (see the "+" signs in the Figure 4). Once deep depletion is established, the remaining unscreened potential drop is accommodated by charge carriers in the valence band, which possess higher mobility and accumulate at the interface in a manner analogous to a metallic surface. As a result, further variations in ionization energy do not significantly alter the Helmholtz potential or the value of κ.

Q7. Achievability of doping concentrations in ultrathin films

Question: In the "conditions of interest" region of Fig. 2c, the doping concentration of the semiconductor thin film is specified to 1017-21 cm−3 under the simultaneous constraint of a thin film

thickness (1-10 nm). To enhance the practical relevance and credibility of your proposed semiconductor design strategy, it would be valuable for the authors to provide illustrative examples and experimental substantiation addressing the achievable doping concentrations of Ni(OH)2, Co(OH)2, FeOOH noted in the study.

Response: We thank the reviewer for pointing out the need to clarify the experimental context and practical relevance of our proposed semiconductor design strategy. We would like first to clarify that the proposed concentration range in our work corresponds to an effective carrier density rather than traditional substitutional bulk doping. This distinction is particularly relevant for materials such as $Ni(OH)_2$, $Co(OH)_2$, and FeOOH, where native defects (e.g., oxygen vacancies, interstitial atoms) and charge carriers play a dominant role in determining electronic properties. In fact, the lower range of the suggested concentration corresponds to the observed range of charge carriers' densities in these materials. But the electronic properties of $Ni(OH)_2$, $Co(OH)_2$, and FeOOH can also be tuned through transition metal doping (e.g., Fe in $Ni(OH)_2$, Ni in $Co(OH)_2$, and Ni in FeOOH)

This electrochemical approach is especially advantageous for electrochemical growth methods, which were employed in the experimental results presented here. Electrochemical deposition allows for the formation of thin film islands with controlled morphology and composition, without requiring precise atomic-level substitutional doping. Such flexibility is crucial for scalable and cost-effective synthesis, as supported by extensive literature precedents. It also enables the tuning of the morphology and effective carrier densities (e.g., through oxygen vacancies) by adjusting electrochemical parameters (e.g., potential, current density, electrolyte composition). For example, Fe-doped $Ni(OH)_2$ films synthesized via electrochemical deposition have achieved effective carrier densities of 10^{18}–10^{21} cm^{-3}, with Fe concentrations up to 16 at.%, as reported in recent studies (Wang, Z et al. 2022. Dealloying-derived Fe-doped Ni (OH) 2/Ni foils as self-supported oxygen evolution reaction catalysts. *J. Energy Mater*, 2, 200019). The method's simplicity and compatibility with industrial processes make it ideal for large-scale applications.

However, there is plenty of other methods to create thin film in more controlled manners. For instance, Pulsed Laser Deposition (PLD) and Chemical Vapor Deposition (CVD) offer atomic-level precision and stoichiometric control, although they are more complex and less scalable than electrochemical methods. PLD, for instance, can produce high-purity films with controlled doping but requires specialized equipment and suffers from slower deposition rates. CVD, on the other hand, provides conformal films but involves toxic precursors and vacuum conditions. Both methods are valuable for fundamental studies and niche applications where precise doping profiles are essential. Other techniques such as Atomic Layer Deposition (ALD) and Ion Beam Sputtering (IBS) can also achieve controlled doping but are limited by slower deposition rates or equipment costs. Chemical Bath Deposition (CBD) offers a low-cost alternative but with less precise doping control. The sol-gel technique is a versatile, wet-chemical method widely used for synthesizing metal oxide and hydroxide thin films, including $Ni(OH)_2$, $Co(OH)_2$, and FeOOH. It is particularly valued for its simplicity, low cost, and ability to produce high-purity, homogeneous materials with controlled composition and morphology. However, an added step is needed to deposit the material in this case, often introducing parasitic contact resistances, which are highly detrimental to electrochemical activity. All of these technics enable doping range of 10^{17}–10^{20} cm^{-3} but it can of course vary from one material to other with specificities we cannot be exhaustive about.

In order to address this point we added a short paragraph in the main p.13 :

"The control of the doping and thickness within the suggested range can be achieved using various techniques (e.g., electrochemical deposition, pulsed laser deposition, chemical vapor deposition, atomic layer deposition, ion beam sputtering, etc.) through traditional substitutional doping but also through

native defects (e.g., oxygen vacancies, interstitial atoms) as it is the case in the experimental results presented here (see Supplementary Note 5)."

As well as a more detailed discussion on the supplementary materials: Supplementary Note 5: Effective carrier density and experimental feasibility of ultrathin semiconductor layers

Response to Reviewer #2

We thank Reviewer #2 for the thoughtful and constructive comments, which help underline the generality of the framework and how much it can extend to other materials and electrochemistry topics. Many of these are yet to explore and we would be happy to work and collaborate on these topics in the future to extend our framework further.

Q1. Applicability to complex and reconstructed catalyst surfaces

Question: Your framework is formulated in terms of work function and Helmholtz potential. How do you expect it to carry over to practical catalyst surfaces such as alloys, high entropy alloys, oxides, and carbon supported catalysts, where reconstruction and partial oxidation under bias are common?

Response: The impact of partial oxidation under bias is particularly interesting to discuss within the framework we developed. An oxidized surface behaves similarly to a metal covered by a thin semiconducting layer, since the passivated surface itself acts as a thin semiconductor. As such, the proposed framework is well suited to describe this situation. Partial surface reconstruction or chemical modification can also alter the electrode surface work function (for metals) or electron affinity (for semiconductors) and therefore the parameters entering the interfacial modeling. Consequently, a proper characterization of the surface chemistry, electronic properties, and their evolution under operating conditions is particularly relevant.

In the case of carbon-supported catalysts, a detailed characterization of the support: its work function, electronic nature (metallic or semiconducting), and surface chemistry, is essential to understand whether surface band bending and a Helmholtz layer form, and how they influence both the catalyst (charge, occupation of orbitals etc) and the electrolyte structure. This understanding is crucial for performance optimization. For instance, the substrate can be engineered to minimize the Helmholtz potential or to increase the entropy of the interfacial electrolyte to favor the reaction, while catalyst properties can be independently tuned for specific reactions.

Similarly, for emerging materials such as alloys, and high-entropy alloys, a systematic characterization of the work function (e.g. by KPFM), surface oxidation (e.g. by XPS), and the properties of the resulting oxide layer such as the ionization energy (e.g. by PYS or UPS) and charge separation (e.g. by SPV) is required to achieve a comprehensive understanding of the interface within the framework presented here.

These perspectives are highly promising and open up a wide range of opportunities for optimization and advanced characterization, which we would be keen to explore in future work. To further emphasize this point, we have added a dedicated comment in the conclusion:

"Partial oxidation, surface reconstruction, and chemical modification under bias naturally fit within the proposed framework, as they effectively modify the electronic parameters governing the interface, such as the work function or electron affinity or even create a semiconductor thin film. This highlights the importance of *operando* characterization of surface chemistry and electronic properties, including for carbon-supported catalysts and emerging materials such as alloys and high-entropy alloys. Such an

approach opens clear pathways for rational interface engineering and performance optimization through simultaneous control of the substrate, catalyst, and electrolyte."

Q2. Extension to reactions beyond HER

Question: Beyond HER, how would you write the same treatment for reactions such as ORR, HOR, or CO2RR? Which steps or intermediates do you expect to be most sensitive to the Helmholtz potential, and how would you implement the surface concentration correction for charged or strongly polar intermediates?

Response: With appropriate caution, the proposed framework can be extended to a broad range of electrochemical reactions. The formation of a Helmholtz potential drop is expected to occur systematically as a consequence of charge equilibration at the interface, largely independent of the specific reaction or electrolyte chemistry, and governed primarily by the electrode work function and the electrolyte chemical potential. This interfacial potential drop in turn controls ion distributions, interfacial electric fields, molecular orientation, electrolyte structuring, and transport properties. In particular, strongly polar electrolytes are expected to be more sensitive to the interfacial electric field, which may significantly affect their rigidity and organization within the Helmholtz layer.

When addressing specific reactions such as the ORR, HOR, or CO_2RR, the exact framework must be adapted to account for the nature of the involved ionic species and their transport through the interfacial layer. For example, in the case of the ORR, which is often diffusion-limited, the experimentally measured half-wave potential should be considered rather than the overpotential, and a diffusion model should be incorporated in addition to the interfacial modeling developed here. Such reaction-specific adaptations of the model are the subject of future work, while the general conceptual framework remains broadly applicable.

To account for this, we added a modification of the main text p.4 and some discussion on the diffusion on the Supplementary Note 0:

"But for large overpotentials, the exponential behavior is limited by charge diffusion in the electrolyte, as observed for the ORR, for instance. A more refined description would involve accounting for mass transport to determine the surface concentration[15] as well as the concentration of reactants adsorbed at the surface, as discussed in Supplementary Note 0. However, such considerations do not affect the general concept presented here, and for simplicity we consider only the surface concentration to be relevant."

Beyond the solvent, strongly polar reaction intermediates are also expected to respond to the interfacial electric field, leading to preferential orientations and potentially stabilizing specific reaction pathways.

Altogether, this interfacial description opens new perspectives not only for electrode optimization, but also for rational electrolyte design and control of reaction selectivity through the properties of intermediates. To highlight these broader implications, we have added a dedicated comment in the conclusion:

"Its presence at the interface arises systematically from charge equilibration and, in turn, governs ion distributions, interfacial electric fields, the orientation of polar intermediates, electrolyte structuring, and transport properties."

Q3. Separation of mass transport and Helmholtz-driven effects

Question: In cases where mass transport limitations are significant, how do you separate diffusion driven concentration polarization from the Helmholtz potential driven surface concentration effect when fitting polarization curves?

Response: In cases where mass transport limitations are significant, it would be necessary to consider the extended version of the Butler–Volmer equation taking into account the local concentration close to the electrode as the "bulk" concentration, utilizing some diffusion model, following the formalism applied in Supplementary Note 1. Then, the gradient of concentration withing the few nm of the Helmholtz layer would be described properly by our framework. It is done implicitly in the case treated here by considering the modified pH at the surface. As to clarify this matter, we decided to explain further in the Supplementary Note 0 and added page 8:

"For diffusion-limited reactions, the "bulk" concentration considered here corresponds to the concentration at the edge of the Helmholtz layer near the surface, which can be determined using mass transport models[15] as explained in Supplementary Note 0, with the same formalism as that developed in Supplementary Note 1."

Q4. Validity of assumptions in non-aqueous or highly concentrated electrolytes

Question: How robust are the thin double layer and linear Helmholtz drop assumptions in non-aqueous electrolytes, ionic liquids, or water in salt systems, where dielectric properties and interfacial structure can differ substantially? What modifications would be needed for those cases?

Response: The framework does not require the electrolyte to be aqueous and can be applied to any electrolyte by accounting for its appropriate chemical potential to determine the Helmholtz potential. The determination of the Helmholtz potential itself is therefore unchanged. However, variations in the dielectric properties of the electrolyte will affect the thickness of the double layer, which is relevant for the determination of κ, while molecular polarity and interfacial interactions influence the rigidity of the electrolyte interface and, consequently, species diffusion. Overall, the general formalism remains valid, but a quantitative application requires proper characterization of the electrolyte properties. To precise this, we added to the conclusion page 19:

"The fundamental role of the Helmholtz potential on charge transfer efficiency extends to any electrochemical reaction in any type of electrolyte."

Q5. Boundary conditions for ultrathin semiconductor layers

Question: For the ultrathin semiconductor layer concept, can you provide more concrete selection criteria and boundary conditions, for example thickness window, band alignment requirements, and the tradeoff between reducing the Helmholtz potential and maintaining access to metallic active sites for different reactions?

Response: Concrete selection criteria and boundary conditions for tuning the parameters of the ultrathin semiconducting layer have been addressed in our responses to Reviewer 1, Questions 5 and 6, as well as Question 7, which discusses the various strategies to optimize layer thickness and doping.

Regarding the trade-off between reducing the Helmholtz potential by introducing a thin hydroxide layer and preserving access to metallic active sites for different reactions, this remains an active and debated research topic within the community, particularly concerning whether the dominant catalytic sites are

located at the interface between semiconducting islands and bare metal regions, or whether significant activity can also occur across the semiconducting islands themselves. While we acknowledge that, in the case of the HER, Pt sites are required to host hydrogen intermediates, our model shows that, for an optimal $Ni(OH)_2$ thickness, the charge carrier density within the $Ni(OH)_2$ layer can be sufficiently high to sustain substantial currents. Koper *et al.* have suggested that the reaction likely occurs well beyond the immediate metal–semiconductor interface, based on initial CO stripping voltammetry and infrared spectroscopy measurements; however, further experimental work is required to resolve this question conclusively. While highly relevant, a detailed discussion of this issue is not straightforward within the present framework and therefore lies beyond the scope of this study. To address this concern, we added a short discussion page 16:

"Additionally, catalytic factors such as the need for exposed platinum sites for reactions involving surface intermediates must also be considered. However, the idea that Ni(OH)2 islands necessarily reduce the electrochemically active surface area of the electrode remains under debate. Koper et al. recently provided initial experimental evidence,[9] based on CO stripping voltammetry and infrared spectroscopy, suggesting a possible direct contribution of Ni(OH)2 to the HER. This interpretation is consistent with our model, which predicts a deep depletion layer in such thin Ni(OH)2 films and, consequently, a high density of charge carriers available to sustain the reaction. Nevertheless, further experimental work is required to conclusively resolve this issue."

Response to Reviewer #3

We thank Reviewer #3 for the detailed and critical assessment. We appreciate the opportunity to clarify the scope of our model.

Q1. Use of proton reduction kinetics in alkaline media

Question: The authors describe HER activity using a Butler-Volmer equation based on the proton reduction reaction, which is appropriate under acidic conditions where protons are readily available at the electrode surface. However, they extend this model to alkaline conditions, where the dominant HER mechanism involves water reduction. This mechanistic discrepancy may be significant, as the kinetics and interfacial phenomena governing HER in alkaline media differ fundamentally from those in acidic environments. Consequently, the direct application of their model to alkaline HER is not justified, casting doubt on the validity of the proposed hypothesis in such conditions.

Response: The question of the consideration of the proton for the reduction reaction we considered rather that water is indeed very pertinent. We chose this case as it is from far the easiest for the reader to understand and the most straightforward. But as the reviewer pointed out it should be questioned in the case of alkaline condition. In that case we can consider the following reaction:

$$\mathrm{H_2O} + e^- = \frac{1}{2}\mathrm{H_2} + \mathrm{OH^-}$$

In contrary to the simple reaction of protons, the reaction of the water molecules will generate OH^- anions whose concentration and accumulation at the surface of the electrode will also be driven by the Helmholtz potential:

$$\frac{[OH^-]_{surf}}{[OH^-]_{bulk}} = e^{+\frac{qV_H}{k_BT}}$$

Positive Helmholtz potential increases the concentration of anions at the surface and prevent the adsorption of water molecules for the reaction happen. Formally, the fraction of occupied sites by water molecules θ_{H_2O} can be described using the Langmuir adsorption description:

$$\theta_{\mathrm{H_2O}} = \frac{K_{\mathrm{H_2O}}[\mathrm{H_2O}]_{surf}}{1 + K_{\mathrm{H_2O}}[\mathrm{H_2O}]_{surf} + K_{\mathrm{H_2}}[\mathrm{H_2}]_{surf} + K_{\mathrm{OH^-}}[\mathrm{OH^-}]_{surf}}$$

With K_{H_2O}, K_{H_2}, and K_{OH^-} the equilibrium constant of adsorption. Initially, the concentration of water molecules at the surface is much greater than the hydroxide concentration and most of the sites are occupied by water molecules. However, while being reduced, hydroxide anions are constantly produced alongside of the hydrogen molecules and compete for the adsorption sites. As an effect, it shifts the equilibrium toward the adsorption of hydroxide anions, effectively reducing K_{H_2O} compared to K_{OH^-}. Furthermore, unlike hydrogen molecules, which desorb and vaporize, hydroxide anions are soluble and accumulates at the surface. $[\mathrm{OH^-}]_{surf}$ can be further enhanced by the Helmholtz potential gradient when the surface of the electrode is charged positively because of their negative charge. All these considerations led to the fraction of the adsorbed molecules to be approximated by:

$$\theta_{\mathrm{H_2O}} \sim \frac{K_{\mathrm{H_2O}}[\mathrm{H_2O}]_{surf}}{K_{\mathrm{OH^-}}[\mathrm{OH^-}]_{surf}}$$

Even if the surface concentration of water molecules is a constant irrespective of the pH or the Helmholtz potential, its proportion adsorbed at the surface of the catalysts is not. It becomes proportional to the inverse of the concentration of hydroxyl ions at the surface which depends on the Helmholtz potential. Thus, the proportion of water adsorbed at the surface in alkaline conditions depends on the Helmholtz potential in the exact same way the proton did in acidic conditions:

$$[\mathrm{H_2O}]_{ad} \propto \theta_{\mathrm{H_2O}} \sim \frac{K_{\mathrm{H_2O}}[\mathrm{H_2O}]_{surf}}{K_{\mathrm{OH^-}}[\mathrm{OH^-}]_{bulk}} e^{-\frac{qV_H}{k_BT}} \propto e^{-\frac{qV_H}{k_BT}}$$

Where $[H_2O]_{ad}$ correspond to the concentration of water molecules adsorbed on reactive sites to be considered for the reaction rate. Considering this expression in the Butler-Volmer equation, one can recognize the exact same dependence on the Helmholtz potential as the one presented in the manuscript.

Note that the same reasoning can be done with the adsorption of protons in the case of acidic condition, but in that case the simplification due to the Helmholtz potential which favors the adsorption of proton simply leads to $\theta_{H^+} \propto [H^+]_{surf}$ hence the adsorbed protons are directly proportional to their concentration at the surface.

As we understand the value of this discussion, we decided to add a complete section Supplementary Note 0 in the supplementary information also discussing the case of diffusion limited reactions.

Q2. Treatment of local pH as a fixed parameter

Question: In their numerical simulation of HER under acidic conditions, the authors fixed the local pH at 10 to illustrate the effect of the Helmholtz potential. However, if the local pH were indeed 10, the HER mechanism would be expected to shift from proton reduction to water reduction, leading to pH-independent kinetics. Such behavior, however, is not observed in the experimental data in acid electrolytes (Nat. Chem. 2013, 5, 300), suggesting that the assumption of a fixed local pH at this value may be flawed. Furthermore, the assumption of a constant local pH contradicts the authors' own

argument that the reduced HER activity arises from a decrease in local proton concentration, which is strongly influenced by the Helmholtz potential. Therefore, the local pH should not be treated as constant but rather as a dynamic variable dependent on the Helmholtz potential. This relationship should be explicitly incorporated into the Butler-Volmer equation used in their model.

Response: As demonstrated in the response to Q1, the fact that the reaction involves water molecules rather than protons does not imply that it is independent of the OH^- concentration at the electrode surface. The reaction kinetics remain governed by the Helmholtz potential and therefore also depend on the electrolyte chemical potential, which is intrinsically linked to the pH.

We emphasize that the pH considered in our framework—used to define the electrolyte chemical potential and to calculate the initial charge transfer and the Helmholtz potential—corresponds to the local pH considered at a few Debye lengths from the electrode surface (i.e., a few to several tens of nanometers). At this proximity to the interface, the local pH is influenced by the ongoing electrochemical reaction and may therefore differ from the macroscopic bulk pH of the electrolyte, as discussed in the manuscript. But it remains "outside" of the double layer and not impacted by the Helmholtz layer.

As correctly pointed out by the reviewer, the ionic concentration gradients established across the electrical double layer by the Helmholtz potential imply spatial variations in proton concentration over nanometer length scales and thus, formally, a variation of pH. However, at such length scales, we deliberately avoid defining pH in the classical thermodynamic sense. Instead, the local proton concentration within the Helmholtz layer is treated explicitly. In this sense, the "local pH" within the Helmholtz layer is not assumed to be constant but is a dynamic quantity governed by the Helmholtz potential, fully consistent with the framework developed in this work.

To address this concern, we added a short discussion page 9 as well as a more extensive discussion in the Supplementary Note 0:

"Between the alkaline and acidic conditions, the chemical potential of the solution is corrected for the local pH close to the surface (at few Debye lengths from the electrode surface) as discussed in the Supplementary Note 0."

Q3. Proton diffusion versus consumption rate

Question: By estimating the proton consumption rate at a current density of -5 mA/cm2, the corresponding rate (j/F) is calculated to be 5.2 * 10^(-8) mol/s/cm2. In comparison, the maximum proton diffusion flux in 0.1 M HClO4—using a proton diffusion coefficient of 9.3 * 10^(-5) cm2/s, a diffusion layer thickness of 0.96 nm (estimated from the Debye length), and calculation by the Fick's 1st law—is approximately 0.097 mol/s/cm2. This diffusion flux is about 1.8 * 10^(6) times greater than the calculated proton consumption rate. Such a large discrepancy indicates that the proton supply far exceeds the consumption, making it numerically implausible to justify the elevated local pH value assumed by the authors. This inconsistency further undermines the validity of their model.

Response: Within the double layer, we do not claim that the concentration is limited by diffusion but simply that it is set by the electrical potential variation. Also, the double layer should not be considered as the proton diffusion layer which is only set by the diffusion and the reaction rate.

Considering a simple steady-state, 1D diffusion normal to a planar electrode, with a constant bulk concentration at infinity, we can determine the profile of concentration over the diffusion layer thickness δ by considering the Fick's 1st law:

$$C(x) = C_\infty - \frac{j}{FD}(\delta - x)$$

Close to the surface, the concentration is thus:

$$C_s = C_\infty - \frac{j}{FD}\delta$$

In the case of large variation of pH we can consider $C_s \ll C_\infty$ so that:

$$\delta \sim \frac{C_\infty FD}{j}$$

Considering $j = 5 \times 10^{-3} A.cm^{-2}$, $D = 9.3 \times 10^{-5} cm^2.s^{-1}$, and $C_\infty = 0.1M = 10^{-4} mol.cm^{-3}$, we can calculate:

$$\delta \sim 1.8\, mm$$

A true steady state is however never really reached; instead, the thickness of the diffusion layer is generally estimated as $\sqrt{D \times t}$, where t is the duration of the experiment. If we consider a CV amplitude of 500 mV and a scan rate of 50 mV/s (as in Markovic's Science paper), then the experiment lasts about 10 s and the diffusion layer thickness is on the order of 300 microns only but remains orders of magnitudes larger than the double layer.

To address this concern, we added a more extensive discussion on the diffusion in the Supplementary Note 0 as well as a modification of the main text p.4:

"But for large overpotentials, the exponential behavior is limited by charge diffusion in the electrolyte, as observed for the ORR, for instance. A more refined description would involve accounting for mass transport to determine the surface concentration[15] as well as the concentration of reactants adsorbed at the surface, as discussed in Supplementary Note 0. However, such considerations do not affect the general concept presented here, and for simplicity we consider only the surface concentration to be relevant."

Conclusion:

We believe that these revisions significantly improve the clarity, rigor, and scope definition of the manuscript. We thank the reviewers again for their valuable input and hope that the revised version will now be suitable for publication.

Sincerely,

Dr. Arsène Chemin
(on behalf of all authors)
